# Foreseeing the future of mutualistic communities beyond collapse

**DOI:** 10.1111/ele.13401

**Published:** 2019-11-10

**Authors:** J. Jelle Lever, Ingrid A. van de Leemput, Els Weinans, Rick Quax, Vasilis Dakos, Egbert H. van Nes, Jordi Bascompte, Marten Scheffer

**Affiliations:** ^1^ Department of Evolutionary Biology and Environmental Studies University of Zurich Winterthurerstrasse 190 CH‐8057 Zurich Switzerland; ^2^ Department of Aquatic Ecology and Water Quality Management Wageningen University P.O. Box 47 NL‐6700 AA Wageningen The Netherlands; ^3^ Computational Science Lab University of Amsterdam NL‐1098 XH Amsterdam The Netherlands; ^4^ Institute of Advanced Studies University of Amsterdam 1012 GC Amsterdam The Netherlands; ^5^ Institut des Sciences de l'Evolution de Montpellier (ISEM) BioDICée Team CNRS Université de Montpellier Montpellier France

**Keywords:** Critical transitions, ecological networks, mutualistic communities, critical slowing down, predictive ecology, forecasting, global environmental change

## Abstract

Changing conditions may lead to sudden shifts in the state of ecosystems when critical thresholds are passed. Some well‐studied drivers of such transitions lead to predictable outcomes such as a turbid lake or a degraded landscape. Many ecosystems are, however, complex systems of many interacting species. While detecting upcoming transitions in such systems is challenging, predicting what comes after a critical transition is terra incognita altogether. The problem is that complex ecosystems may shift to many different, alternative states. Whether an impending transition has minor, positive or catastrophic effects is thus unclear. Some systems may, however, behave more predictably than others. The dynamics of mutualistic communities can be expected to be relatively simple, because delayed negative feedbacks leading to oscillatory or other complex dynamics are weak. Here, we address the question of whether this relative simplicity allows us to foresee a community's future state. As a case study, we use a model of a bipartite mutualistic network and show that a network's post‐transition state is indicated by the way in which a system recovers from minor disturbances. Similar results obtained with a unipartite model of facilitation suggest that our results are of relevance to a wide range of mutualistic systems.

## Graphical abstract

While detecting upcoming critical transitions in complex ecosystems is challenging, predicting what comes after a critical transition is terra incognita altogether. Here, we address the question of whether the relative simplicity of the dynamics of complex mutualistic communities allows us to foresee a community's future state.

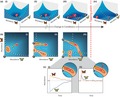

## INTRODUCTION

Empirical studies of lakes, arid ecosystems, coral reefs and tropical forests suggest that remarkably sudden transitions to alternative stable states may occur when changing environmental conditions pass a critical value (Scheffer *et al.* 1993; Rietkerk & Van de Koppel 1997; Scheffer *et al.* 2001; Hirota *et al.* 2011). While the outcome of such transitions is relatively predictable when a few leading species or species groups determine the state of an ecosystem, this may not be the case when ecosystem dynamics are determined by many interacting species. Species traits as well as their sensitivity to changing conditions are known to be highly diverse, and many drivers of environmental change are known to have multiple simultaneous effects on species communities. A change in climate may, for example, affect the distribution, phenology, physiology, behaviour and relative abundances of species, and these changes may, in turn, affect the strengths of interactions between species (Kareiva *et al.* 1993; Memmott *et al.* 2007; Suttle *et al.* 2007; Tylianakis *et al.* 2008; Burkle *et al.* 2013; Høye *et al.* 2013; Usinowicz & Levine 2018). The specific ways in which interactions are arranged in complex ecological networks are known to be crucial for the stability of ecosystems (Kareiva *et al.* 1993; De Ruiter *et al.* 1995; McCann 2000; Sole & Montoya 2001; Neutel *et al.* 2002; Montoya *et al.* 2006; Bastolla *et al.* 2009; Rohr *et al.* 2014). Gradual changes in these patterns and other complex simultaneous effects of changing environmental conditions may therefore lead to regime shifts of which the outcomes are highly unpredictable (Scheffer *et al.,* 2012).

The response of ecosystems to a change in environmental conditions is determined by the relative strengths of positive and negative feedback loops in the networks of interactions between species or between species and their environment. Immediate negative feedbacks (e.g. due to intraspecific competition) have stabilising effects, while positive or ‘reinforcing' feedbacks are destabilising and a necessary condition for the existence of alternative stable states (Thomas 1981; Snoussi 1998; Gouzé 1998). Critical transitions towards such states may occur when changing conditions alter a system's feedbacks such that destabilising, positive feedbacks gain in strength relative to stabilising, immediate negative feedbacks. A classic example is found in shallow lakes where an increase in algae leads to an increased turbidity and the suppression of aquatic plants. As a consequence, more nutrients become available to algae which enhances algae growth. A clear‐water, plant‐dominated state may therefore switch to a turbid, algae‐dominated state when gradually increasing nutrient levels pass a critical value. Recovery from such transitions requires a relatively large reduction in nutrient availability, a phenomenon called ‘hysteresis’ (Scheffer *et al.* 1993). Other examples of such switching behaviour are found in coral reefs, woodlands, deserts, and oceans (May 1977; Wilson & Agnew 1992; Scheffer *et al.* 2001), as well as in many other systems such as the climate (Hare & Mantua 2000; Scheffer *et al.* 2001; Clark *et al.* 2002; Alley *et al.* 2003; Lenton *et al.* 2008), the economy (Diamond & Dybvig 1983; Arthur 1989; Easley & Kleinberg 2010), and human cells (Hasty *et al.* 2002; Ferrell 2002; Lee *et al.* 2002; Tyson *et al.* 2003; Angeli *et al.* 2004).

Mutually beneficial interactions are, perhaps, the most intuitive examples of positive feedback loops in complex ecological networks, metapopulations or other complex environmental systems. Previous studies have emphasised the importance of such interactions in communities of flowering plants and animal pollinators or seed dispersers (Jordano, 1987; Bascompte *et al.,* 2003). Mutually beneficial interactions between zooxanthellae, coral species and invertebrates occur in coral reefs where a diversity of coral species provides food, shelter, and reproduction sites for other organisms (Moberg & Folke 1999; Wilson *et al.* 2006; Stella *et al.* 2011). Nutrient exchange with mycorrhizal fungi and nitrogen‐fixing bacteria is fundamental for plant communities (Kiers *et al.* 2011) and mutualistic interactions are of importance for microbial communities where multiple species are involved in the degradation of organic substrates (Schink 2002; Stolyar *et al.* 2007). Indirect facilitation may occur between plant species when modifying harsh environments (Wilson & Agnew 1992; Callaway 1995; Holmgren *et al.* 1997; Rietkerk *et al.* 2004) and the exchange of individuals between habitat patches may be fundamental for metapopulations (Hanski 1998). Previous work suggested that critical transitions may occur due to the positive feedback resulting from such mutually beneficial relationships in plant–pollinator communities because a decline in pollinator abundances may negatively affect plant abundances, which in turn is bad for pollinators (Lever *et al.* 2014). Similar transitions may occur in metapopulations due to a ‘rescue effect’ (Hanski 1998) and in facilitative communities due to an ‘Allee effect’ (Courchamp *et al.* 1999; Stephens *et al.* 1999; Rietkerk *et al.* 2004). The observation that the relative strength of facilitative interactions tends to increase with environmental stress (Bertness & Callaway 1994; Maestre *et al.* 2009; Tur *et al.* 2016) suggests that competitive communities may become increasingly mutualistic as conditions change. The aforementioned positive feedbacks and associated critical transitions may thus also occur in communities where mutually beneficial interactions were not particularly strong under more advantageous conditions.

Here, we propose a new class of indicators that may allow us to detect the specific way in which species are affected by an increase in the relative strength of a positive feedback prior to a critical transition. The essence of our approach is that we seek the direction in a system's phase space (i.e. a multidimensional space in which each axis corresponds to the abundance of a species) in which a system becomes increasingly sensitive to small subcritical disturbances. Earlier studies have shown that an increasingly slow recovery from small disturbances may be indicative of a loss of resilience prior to critical transitions (Wissel 1984; Van Nes & Scheffer 2007). Various indicators of this phenomenon known as ‘critical slowing down’ may therefore serve to detect an increase in the likelihood of critical transitions (Scheffer *et al.* 2009; Dakos *et al.* 2012). Here, we take advantage of the fact that resilience is not lost equally in all directions. Disturbances have a size (i.e. the total amount of change) and a direction (i.e. the relative amount of change in each species). The more similar a disturbance's direction to the direction in which increasingly small perturbations may cause critical transitions, the stronger the effect of critical slowing down. Provided that there are no oscillatory, chaotic or other complex dynamics, a system's future state will most likely lie in the same approximate direction.

**Figure 1:**
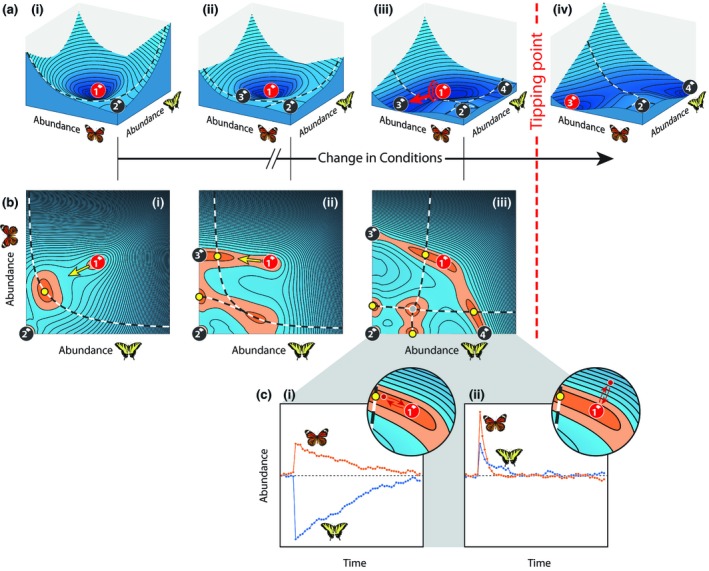
Stability properties for a small network of two pollinators (shown) and two plants (not shown). (a) Attraction basins (valleys) of alternative stable states (balls) are separated by thresholds (dashed curves). Initially, the only alternative to pristine state 1 is fully collapsed state 2 (a.I). When conditions change, two additional, partially collapsed states appear (states 3 and 4). The initial, pristine state loses resilience after state 3 appears (a.II and a.III). Eventually, the threshold towards state 3 approaches the pristine state so closely that a critical transition towards this state becomes inevitable (a.III and a.IV). (b) Alternative stable states, saddle points (yellow dots) and hilltops (grey dots) are surrounded by areas in which the landscape's slope, and thus the rate at which abundances change, is nearly zero (indicated in orange). Higher speeds are found further away from these points. The direction of slowest recovery changes substantially before future state 3 appears (yellow arrow, b.I and b.II). After state 3 appears, the system slows down in the direction of the saddle point on the approaching threshold (b.II and b.III). (c) Slow recovery from a perturbation towards the saddle point (c.I) as opposed to the much faster recovery from an equally large perturbation in another direction (c.II).

To get an intuitive understanding of the principle behind our approach, consider a small plant–pollinator community of which the dynamics can be represented by a landscape of valleys, hills and ridges (Fig. 1a and Appendix S2 in Supporting Information). In this landscape, every possible combination of pollinator abundances is represented by a unique point, while the speed and direction in which abundances change corresponds roughly to the slope of the landscape. The lowest points of the landscape's valleys or ‘attraction basins’ represent alternative stable states. As conditions change, the shape of the landscape changes and new basins appear. When a threshold comes close to the network's initial state, a small perturbation in the right direction can invoke a transition into another attraction basin. Eventually, the basin around the network's initial state disappears altogether and the system inevitably shifts into one of the alternative basins. The question we ask is whether we may know beforehand to which of the alternative attractors a system will most likely to shift. The clue is that the slope of the initial state's attraction basin changes in a characteristic way before the transition occurs. A ‘mountain pass’ towards the system's future state is formed, marked by a ‘saddle point’ in the landscape. The initial state's attraction basin becomes increasingly shallow in the direction of this pass and the recovery from perturbations increasingly slow (Fig. 1b, c; Fig. S2). This direction is what we refer to as the ‘direction of critical slowing down’ and is indicative of the relative gain or loss in abundance of each species after an impending critical transition.

To explore whether the direction of critical slowing down might be indicative of the future state of mutualistic communities, we use a model of a bipartite mutualistic network in which critical transitions are known to occur (Lever *et al.* 2014; Dakos & Bascompte 2014; Jiang *et al.* 2018). This model was originally developed to describe the interactions between flowering plants and animal pollinators or seed dispersers (Bastolla *et al.* 2009), but may describe any system characterised by competition within and cooperation between species groups. Previous work has shown that indirect facilitation occurs between pollinators when they interact with the same plant species (Moeller 2004; Ghazoul 2006; Bastolla *et al.* 2009). This indirect facilitation makes a network more resilient (i.e. the minimum size of perturbations or the amount of change in environmental conditions needed to cause a critical transition is larger). When pollinators continue to facilitate each other under increasingly harsh environmental conditions they may, however, also collapse simultaneously because they depend on each other for survival (Lever *et al.* 2014).

We generate time series in which the resilience of a network's initial state is gradually undermined by altering the relative strength of mutualistic interactions. Oscillatory or other complex dynamics occurring after a threshold is passed may negatively affect the performance of the here proposed class of indicators but are unlikely in purely mutualistic systems (i.e. systems in which all interspecific interactions are positive) because they require at least one delayed negative feedback (i.e. a negative feedback with a time lag) usually occurring as the result of an uneven number of negative interactions in feedback loops of two or more species (Levins 1974; Thomas 1981; Puccia & Levins 1985; Hastings & Powell 1991; Goldbeter 1996; Snoussi 1998; Gouzé 1998; McCann *et al.* 1998; Dambacher *et al.* 2003). Few real ecosystems can, however, be expected to be purely mutualistic. Different scenarios are therefore explored, varying from a scenario where positive feedbacks are the only cause of instability (i.e. in purely mutualistic systems) to scenarios in which the destabilising effects of delayed negative feedbacks are stronger (i.e. in mixed systems with mutualistic and competitive interactions). To determine the direction of critical slowing down, we study changes in the fluctuations around the species' mean abundances and determine whether they can be used to predict a network's post‐transition state. To explore whether the results obtained with this model may hold for a wider class of mutualistic systems, we investigate whether similar results are obtained with a more general, unipartite model of competition and facilitation between species.

## COMMUNITY MODEL

We use a dynamic model describing the interactions between two types of species: plants (*P*) and pollinators (*A*). As in Bastolla *et al.* (2009), species of the same type compete with each other, while species belonging to a different type interact mutualistically. The dynamics of species *i* belonging to a group of *S* ^(^ *^A^* ^)^ pollinator species are as follows:

(1) $$\frac{dN_{i}^{\left(A\right)}}{\mathrm{dt}}=\frac{R_{i}\left(N^{\left(P\right)}\right)}{1+h_{i}R_{i}\left(N^{\left(P\right)}\right)}N_{i}^{\left(A\right)}-\sum_{j=1}^{S^{\left(A\right)}}c_{\mathrm{ij}}N_{j}^{\left(A\right)}N_{i}^{\left(A\right)}-d_{i}N_{i}^{\left(A\right)}+\epsilon_{i}.$$

Plant dynamics are described by a similar formula, which can be found by exchanging indices *A* and *P*. Unless stated otherwise, this procedure can be applied to all formulas in this paper.

Species *i* has abundance *N_i_*, which may increase due to mutualistic interactions with members of the other species type. The rate at which the abundance of species *i* increases depends on the total amount of resources provided by mutualistic partners, $R_{i}\left(N^{\left(P\right)}\right)$ (i.e. nectar for pollinators and pollen for plants). As in Okuyama & Holland (2008) and Bastolla *et al.* (2009), we assume that species are limited in their capacity to process resources and become saturated when the amount of resources provided is high. The rate at which species become saturated is determined by saturation term *h_i_*. The total mutualistic benefit, $R_{i}\left(N^{\left(P\right)}\right)$, depends on the abundance of mutualistic partners as follows:

(2) $$R_{i}\left(N^{\left(P\right)}\right)=\sum_{k=1}^{S^{\left(P\right)}}\gamma_{\mathrm{ik}}N_{k}^{\left(P\right)},\;$$

where $\gamma_{\mathrm{ik}}$ is the mutualistic interaction strength (i.e. the rate at which resources become available to species *i*) due to its interaction with species *k*.

Species of the same type compete directly amongst each other (e.g. plants for soil nutrients and pollinators for nesting sites). Intraspecific competition, *c_ii_*, is assumed to be substantially stronger than interspecific competition, *c_ij_*, such that species do not easily outcompete each other. Independent of mutualistic and competitive interactions, several processes may simultaneously enhance or reduce population growth. We assume that the combined effect of these processes is negative, which is incorporated by mortality rate *d_i_*.

Species experience small stochastic perturbations incorporated through noise term $\epsilon_{i}$:

(3) $$\epsilon_{i}=\delta_{i}\frac{\mathrm{dW}}{\mathrm{dt}}.$$

$\epsilon_{i}$ fluctuates in time due to a Wiener process, *W*, with mean zero and standard deviation $\delta_{i}$. The Wiener process is a continuous‐time stochastic process generating white noise. To prevent noise leading to negative abundances, we assume that dN/dt=0 when *N *< 0.001.

### Coexistence and relative mutualistic benefits

As the number of species and/or the strength of interspecific competition increases, it becomes increasingly difficult to assign parameters such that all species may stably coexist. In previous work, a trade‐off was assumed between the number and the strength of mutualistic interactions which prevented species with many interactions from becoming overly abundant and outcompeting other species (Bastolla *et al.* 2009; Lever *et al.* 2014; Dakos & Bascompte 2014; Jiang *et al.* 2018). Here, we assume mutualistic interaction strengths to vary continuously (i.e. pollinators may interact with all plant species and vice versa) which allows us to explore gradual changes in interaction structure beyond the fixed structure of a predefined mutualistic network. A different kind of balancing relationship is therefore required, and mutualistic interaction strengths, $\gamma_{\mathrm{ik}}$, are determined as follows:

(4) $$\gamma_{\mathrm{ik}}=\frac{\theta_{\mathrm{ik}}R_{i}\left({\hat{N}}^{\left(P\right)}\right)}{{\hat{N}}_{k}^{\left(P\right)}},$$

in which the relative mutualistic benefit, $\theta_{\mathrm{ik}}$, corresponds to the fraction of the total amount of resources provided by species *k*, and $R_{i}\left({\hat{N}}^{\left(P\right)}\right)$ to the total amount of resources received by species *i* at the system's non‐trivial equilibrium (i.e. the equilibrium point at which all species have a non‐zero abundance). There are different costs and benefits associated to different feeding strategies, for example being a specialist or a generalist, or interacting with specialists or generalists (Morales & Traveset, 2008; Tur *et al.,* 2016). This way of assigning mutualistic interaction strengths makes sure that a species' total amount of resources received is independent from a species' relative feeding preferences (i.e. we assume the sum of these costs and benefits to be approximately the same for each strategy). The sum of a species' relative mutualistic benefits, $\theta_{\mathrm{ik}}$, is one. A change in relative mutualistic benefits thus does not affect the equilibrium abundances of species, because the total amount of resources provided to each species remains the same (see Appendix S5).

### Changing environmental conditions and the direction in which resilience is lost

To test whether the direction of critical slowing down is indicative of a system's future state, we study our ability to predict a system's future state when changing conditions lead to substantial changes in the strength of positive feedbacks and the direction in which they have destabilising effects. Such changes may occur when changing conditions fundamentally alter the ways in which species relate to each other.

Positive feedbacks and the direction in which resilience is lost can be studied when determining the elements of the Jacobian matrix at a system's non‐trivial equilibrium. Each element in this matrix describes how a change in the abundance of species *i* affects the growth of species *j*, $dN_{j}/dt$. At a tipping point, the dominant eigenvalue of the Jacobian matrix is zero and the slope of the direction in which a system recovers slowest from perturbations is indicated by the eigenvector corresponding to this eigenvalue. The strength of the positive feedback between pollinator *i* and plant *j* can be determined by multiplying the Jacobian's off‐diagonal elements $\alpha_{\mathrm{ij}}*\alpha_{\mathrm{ji}}$. In a two‐species system, a tipping point is reached when the strength of this feedback is equal to the multiplication of the two direct negative feedbacks $\alpha_{\mathrm{ii}}*\alpha_{\mathrm{jj}}$. Similar relationships can be obtained when studying larger systems (Levins 1974; Thomas 1981; Puccia & Levins 1985; De Ruiter *et al.* 1995; Goldbeter 1996; Snoussi 1998; Gouzé 1998; Neutel *et al.* 2002; Dambacher *et al.* 2003; Neutel & Thorne 2014).

Some species contribute more to the instability caused by positive feedbacks than others. The effect of a temporary change in the abundance of mutualistic partners, as described by the Jacobian matrix, for example, is small when species are highly saturated (i.e. $R_{i}\left({\hat{N}}^{\left(P\right)}\right)$ and/or *h_i_* is large). Positive feedbacks are therefore weak and the resilience of the here studied networks is high when relative mutualistic benefits, $\theta_{\mathrm{ik}}$, are distributed such that most resources are obtained from the same, highly saturated species (see Appendix S1 and Fig. S1). In more complex communities, such a distribution resembles a nested structure as is commonly observed in mutualistic networks, as in those networks species tend to obtain resources from the same mutualistic partners as well (Bascompte *et al.* 2003 and Fig. S6). The interrelationships between saturated and non‐saturated species are asymmetrical as in Bascompte *et al.* (2006).

As a starting point for further research, we explore a scenario in which a change in the aforementioned distribution of relative mutualistic benefits, $\theta_{\mathrm{ik}}$, undermines the resilience of the mutualistic networks while keeping all other properties (e.g. non‐trivial equilibrium abundances and the negative effects of inter‐ and intraspecific competition) constant (see Appendix S5). Increasingly strong positive feedbacks emerge when two or more non‐saturated species start to interact increasingly strongly with each other. Eventually, this will lead to a full or partial network collapse depending on the specific way in which relative mutualistic benefits are changed. Conditions, *M*, affect relative mutualistic benefits as follows:

(5) $$\theta_{\mathrm{ik}}^{*}=\theta_{0,ik}+\left(\theta_{final,ik}-\theta_{0,ik}\right)M,$$

where $\theta_{0,ik}$ is the initial, $\theta_{final,ik}$ the final and $\theta_{\mathrm{ik}}^{*}$ the actual relative mutualistic benefit. Conditions, *M*, change from zero to one over time, *t*, such that dM/dt=1/T, where *T* is the total simulation time. Mutualistic interaction strengths, $\gamma_{\mathrm{ik}}$, are updated as described in equation 4. The species and interactions involved in the positive feedback leading to a critical transition, the direction in which this feedback amplifies change and the nature of a system's future state are determined by the specific way in which interactions are altered.

In addition to the scenario in which only the relative mutualistic benefits change, we explore scenarios in which the non‐trivial equilibrium abundances of species change as well due to a change in the total amount of resources received from mutualistic partners (see Appendix S5).

### Determining the direction of critical slowing down

Although measuring the recovery rate from experimental perturbations is the most direct way to determine the direction of critical slowing down, an experimental approach may be impractical or even impossible when studying complex networks. The development of alternative methods to determine the direction of critical slowing down is therefore of importance. Previous studies suggested that small changes in the statistical properties of time series, for example an increase in variance, autocorrelation, skewness and spatial correlation, may be used as an indicator of a change in the proximity to a tipping point (Scheffer *et al.* 2009; Dakos *et al.* 2012). Here, we explore whether changes in the statistical properties of time series may be used to predict the future state of mutualistic communities.

When assuming a continuous regime of random perturbations, a system will spend most time away from its equilibrium state in the direction in which it recovers slowest from perturbations (see Appendix S2). When approaching a tipping point, the distribution of natural fluctuations around the species mean abundances should therefore become increasingly elongated in the direction in which a system slows down (Fig. S3). To detect such change, we analyse our model‐generated times series by determining the direction and magnitude of such asymmetry in a rolling window. This window has a fixed size and is moved along the time series as new data become available. To determine the direction in which abundances are distributed asymmetrically, we use a principal component analysis of which the first principal component corresponds to the line in the network's phase space along which variance is highest (see Held & Kleinen 2004; Chen *et al.* 2012; Suweis & D’Odorico 2014; Dakos 2018 and Chen *et al.* 2019 for related approaches). Abundances are distributed asymmetrically either in an up‐ or downward direction along this component. To determine the direction of our indicator, we orthogonally project the time series on the first principal component and determine the direction in which the projected time points are skewed (Fig. S4a–e). The magnitude of the indicator is determined by the fraction of the total variance explained by the first principal component. This direction and magnitude together form a vector which is our indicator of a network's future state (Fig. S4f).

A network's phase space has as many axes as there are nodes in a network. Our indicator thus has multiple components; one for each species (Fig. S4f). Each component, or ‘score on the indicator’, gives an indication of the extent and direction in which the abundance of each individual species is distributed asymmetrically. The indicator accurately points towards the future state when its components, or ‘scores’, are directly proportional to the difference in abundance between a network's initial and future state. Species with a negative score are expected to decrease, while species with a positive score are expected to increase. Species with a relatively large score are expected to change more in abundance than species with a comparably smaller score. An increase in the indicator's magnitude is reflected by more extreme (positive or negative) scores.

To assess the quality of the prediction, we determine the angle between the indicator's slope, as determined by the first principal component, and the direction of the observed shift in abundance. As a measure of similarity, we take one minus the probability that the angle between two unrelated, random vectors is smaller (see Appendix S3). We consider the indicator's slope to be accurate when this measure of similarity is above 0.99. When time points are also skewed towards a network's future state, we consider the prediction to be fully accurate.

### Simulations and parameter settings

We analyse several data sets consisting of 1000 model‐generated time series in which the above described mutualistic networks approach a tipping point. For each time series, we compute the change in direction and magnitude of the indicator on the pollinator abundances (see Appendix S4). The distribution from which interspecific competitive interaction strengths are sampled, the number of plant and pollinator species, and the way in which changing conditions affect non‐trivial equilibrium abundances differ among data sets (see Appendix S5). The resilience of mutualistic networks is, in all cases, undermined by a change in the distribution of mutualistic benefits leading to a substantial increase in the relative strength of positive feedbacks or delayed negative feedbacks. Declining abundances may have an additional negative effect on resilience.

To explore the effects of oscillatory, chaotic or other complex dynamics, we analyse data sets of which the strength and variability in interspecific competitive interaction strengths, *c_ij_*, varies. Delayed negative feedbacks become stronger as the strength and variability of interspecific competition increases. To provide a clue as to how (un)likely it is to find transitions to oscillatory, chaotic or other complex dynamics, we determine for each time series whether the system approaches a Hopf or a saddle‐node bifurcation.

Networks were discarded from a data set when they were unstable at initial conditions, *M = 0*. We determined the frequency at which this occurred as a measure of how difficult it is to find a stable solution. The final distribution of relative mutualistic benefits, $\theta_{final,ik}$, was redrawn either when a network would become unstable within the range of conditions *M *= (0,0.5), or when a network would still be stable at *M = 1*.

### A more general, unipartite model of competition and facilitation

To explore whether the indicator may work for a wider class of systems, we investigate whether similar results are obtained with a more general model of competition and facilitation. The positive feedback between plants and pollinators in the previously described communities can be seen as an Allee effect (i.e. a positive relationship between the growth and density of populations, see Courchamp *et al.* 1999 and Stephens *et al.* 1999). The indirect facilitation occurring between pollinators when interacting with the same plant species is not fundamentally different from the facilitation occurring between plant species when ameliorating the same harsh environment, or other forms of interspecific facilitation occurring in ecosystems. The most essential properties of a group of pollinator species may therefore be captured as follows:

(6) $$\frac{dN_{i}}{\mathrm{dt}}=r_{i}N_{i}\left(\frac{\sum_{j=1}^{S}\gamma_{\mathrm{ij}}N_{j}}{A_{i}}-1\right)\left(1-\frac{\sum_{j=1}^{S}c_{\mathrm{ij}}N_{j}}{K_{i}}\right)-d_{i}N_{i}+\epsilon_{i},$$

where *N_i_* is the abundance of species *i*. When the abundances of other species and mortality rates, *d_i_*, are zero, species may grow in abundance until they reach carrying capacity *K_i_*, or collapse to extinction when abundances are below critical abundance *A_i_*. The speed at which species abundances change is determined by growth rate *r_i_*. Facilitation is mediated by facilitation rate $\gamma_{\mathrm{ij}}$. Strong interspecific facilitation allows species to recover from large disturbances (i.e. below critical abundance *A_i_*). Species with a high critical abundance *A_i_* depend strongly on this facilitation, and a community's overall resilience is highest when such species are facilitated relatively strongly by species with a low *A_i_*. The relative strength of interspecific competition is determined by *c_ij_*. Other causes of abundance loss are incorporated through mortality rate *d_i_*. Species are assumed to experience small stochastic perturbations, as in the bipartite mutualistic network model, through noise term $\epsilon_{i}.$

The main difference between the here presented model and the previously described plant‐pollinator model is that it is a unipartite model describing one set of interacting species. The means by which facilitation occurs are, in contrast to the above described plant‐pollinator model, not explicitly described. Parameter settings and results can be found in Appendix S6 and S7.

## RESULTS

**Figure 2:**
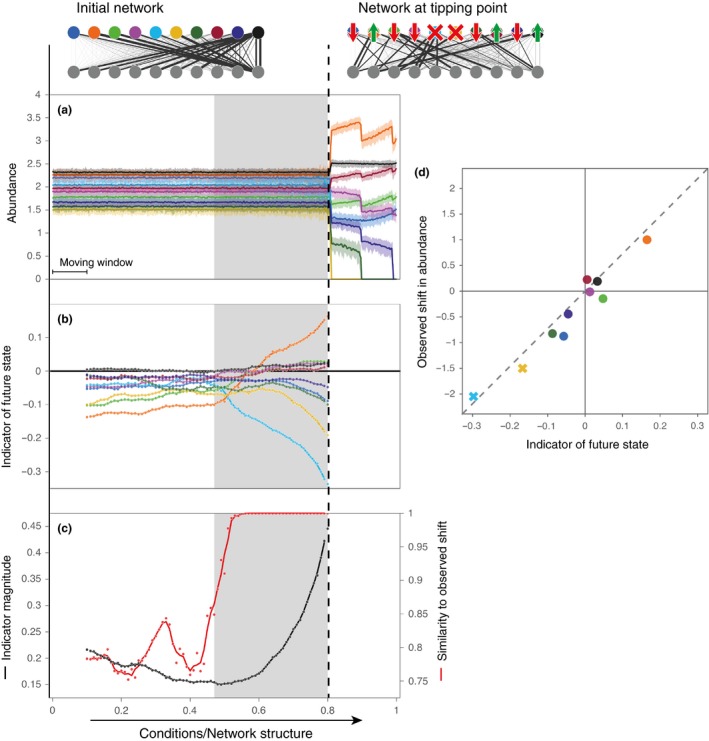
Directional slowing down in a mutualistic network as detected by our indicator. (a) Time series of species belonging to one part of a bipartite mutualistic network (i.e. the pollinators). At the tipping point two species collapse to extinction (light blue and yellow). (b) The indicator of the future state measuring the direction in which fluctuations are distributed asymmetrically. Scores on the indicator indicate the relative predicted gain or loss of each node. (c) The magnitude of the indicator, reflecting the extent to which fluctuations are distributed asymmetrically, plotted together with the accuracy measured as the similarity between its direction and the observed shift in abundance. Grey bands indicate the period in which the indicator's magnitude increases significantly. This period likely corresponds to the period in which the network rapidly loses resilience (as in Fig. 1a.II and 1a.III). The accuracy increases rapidly at the beginning of this period. (d) The observed changes in abundance versus the scores on the indicator just before the tipping point. Extinct species are indicated with crosses. The observed shift is nearly proportional to the scores on the indicator as points are close to a straight line through the origin.

We found that, when interspecific competitive interaction strengths are weak, instability nearly always arises from the positive feedback between plants and pollinators or from the Allee effect in the above described mutualistic or facilitative communities. Instability is caused by a saddle point approaching the communities' initial state and at least one species will collapse to extinction when a tipping point is passed. Other species may either gain or lose in abundance depending on the communities' initial properties and the way in which they are affected by changing environmental conditions (Fig. 2a). Critical transitions were nearly always preceded by a period in which the indicator's magnitude would increase significantly indicating that the distribution of fluctuating species abundances becomes increasingly asymmetric (see Appendix S7, Fig. 2b–d and Fig. S7–S9). As with the small mutualistic network in Fig. 1, the indicated direction typically shifts towards a system's future state at the beginning of this period. The indicator thus consistently pointed towards a community's future state while increasing in magnitude prior to a critical transition, when interspecific competitive interactions were weak.

**Figure 3:**
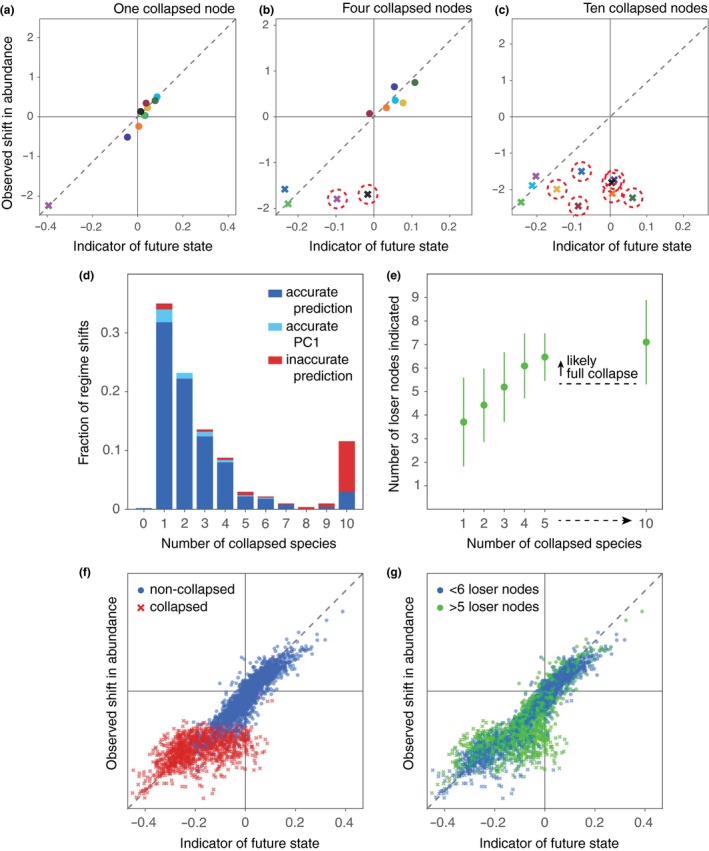
Cascading collapses and the indicator's performance when predicting the future state of mutualistic networks. (a–c) Examples of the relationship between the scores on the indicator and the observed shifts in abundance when a single, when four, and when all pollinator species collapse to extinction. The change in abundance of winners, losers and two or three collapsed species was almost always accurately indicated. The loss in abundance of additional species collapsing (red circles) was underestimated. (d) The fraction of regime shifts after which a certain number species collapsed to extinction. The fraction for which the change in abundance was not accurately indicated is shown in red. Inaccurate predictions (as in panel c) usually occurred prior to a full network collapse. (e) Relationship between the number of species collapsing and the number of species with a negative score on the indicator (mean and SD). When the number of species indicated to lose in abundance was high, we were often dealing with a full network collapse. (f–g) Combined plots of the 900 best indicated transitions in a data set of 1000 regime shifts. Species remaining after a regime shift (blue dots, panel f) are indicated more accurately than collapsing species (red crosses, panel f). Species of which the loss in abundance prior to a collapse was underestimated usually belonged to networks of which 5 or more species were indicated to lose in abundance (green dots and crosses, panel g). Competitive interaction strengths were taken from a low to intermediate range (i.e. 0.02–0.08).

A notable exception to this general pattern occurred when competitive interaction strengths were taken from a low to intermediate range (e.g. ∼U(0.02,0.08)). We found that, for such a range, full network collapses were not always indicated accurately. Transitions would lead either to the collapse of relatively few species or to a collapse of the entire network (Fig. S9). Both the inaccurate prediction of full network collapses and the absence of intermediate‐size, partial network collapses may occur because critical transitions lead to a series of cascading, partial network collapses. The likelihood of an additional collapse increases as more species collapse (Solé & Montoya 2001; Memmott *et al.* 2004; Rezende *et al.* 2007). The most likely outcome of a series of cascading, partial network collapses is therefore a collapse of the entire network. In such a scenario, the indicator will accurately indicate the initial regime shift but will not foresee the cascade of partial network collapses immediately following it (Fig. 3). In some time series, we observed that regime shifts consisted of several consecutive collapses (Fig. S10a, b). The amount of time in between two consecutive collapses can, however, be extremely small. Also when cascades were not clearly visible, we suspect therefore that the inaccurate prediction of a full network collapse is caused by a cascading collapse.

Cascading, full network collapses were uncommon when interspecific competitive interaction strengths were drawn from other ranges (Fig. S9). When there is no competition between species, full network collapses are very common, well indicated and do not show signs of being caused by a cascade of partial network collapses (as in Fig. S10c). When competitive interactions are strong, few species tend to collapse to extinction, while most or all other species gain in abundance from a transition. Apart from the specific range from which competitive interaction strengths were drawn, cascading collapses were found to become increasingly common when the noise level increases suggesting that they, in part, result from a low resilience of a system's future state (Fig. S11, S12). A relatively large number of species was usually indicated to lose in abundance when a, likely, cascading collapse occurred (e.g. 7 out of 10 on average, Fig. 3e). As an alternative indicator of the likelihood of a cascading, full network collapse we propose therefore to use the number of species indicated to lose in abundance.

**Figure 4:**
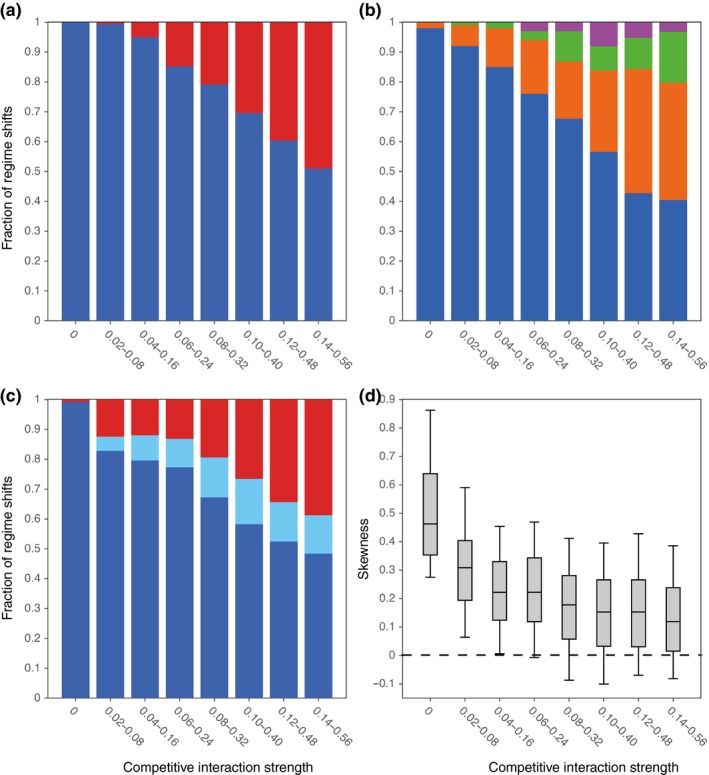
Hopf bifurcations and the predictability of a network's future state when sampling competitive interaction strengths from different parameter ranges (ranges are indicated on the x‐axis). As the strength and variability of competition increases, Hopf bifurcations become increasingly frequent as well as the number of networks of which the future state is determined by chance. (a) The frequency of saddle‐node (blue) and Hopf bifurcations (red) for different data sets. A high frequency of Hopf bifurcations indicates that transitions towards oscillatory, chaotic or other complex dynamics are common. (b) The fraction of cases in which, after five simulations in which a network's resilience was undermined in the exact same way, a network would always shift to the same state (blue), to one out of two states (orange), to one out of three states (green) or to one of four or more potential future states (purple). (c) The fraction of accurately indicated regime shifts (dark blue), the fraction accurately indicated by the first principal component (i.e. the slope of the indicator is accurate) but not by the direction in which time points are skewed (light blue), and the fraction of inaccurately indicated regime shifts (red). (d) The skewness of time points projected on the first principal component. A positive skewness means that time points are skewed in the direction of a network's future state. The skewness is shown for regime shifts that were accurately indicated by the first principal component.

As the strength and variability of interspecific competition increases, Hopf bifurcations, leading to oscillatory, chaotic or other complex dynamics, become increasingly common. After such transitions, the system remains highly sensitive to small‐scale stochastic perturbations and may end up in any of several potential future states (Fig. 4a, b, and Fig. S13–S15). To which of these states a system will shift is determined by chance and thus hard to predict. For the highest competition level we tested, we found that such hard‐to‐predict regime shifts made up about 60% of a data set. Higher levels were not tested because, as the strength of competition increases, it becomes increasingly difficult to generate networks of which the initial, non‐trivial state is stable. More specifically, we found that the probability of a network to be stable at initial conditions, *M = *0, is nearly one when interspecific competitive interaction strengths were taken from the aforementioned lower ranges and below 0.01 when they were taken from the highest here reported range (Fig. S16). The indicator accurately indicated about 50% of the regime shifts in this ‘worst‐case scenario’ (some of the hard‐to‐predict regime shifts were indicated accurately). When there is no competition between species, this percentage was nearly 100% (Fig. 4c, d).

Qualitatively similar results were found when, in addition to a change in relative mutualistic benefits, the species' non‐trivial equilibrium abundances changed as well (see Appendix S7 and Fig. S17). Full network collapses are more frequent when abundances tend to decrease and the period in which the indicator's magnitude increases prior to a critical transition tends to be somewhat shorter when abundances change over time. Examples in Fig. S18–S20 suggest that the direction of the first principal component is initially determined by the way in which abundances change over time. It may, therefore, take longer before the direction in which abundances are distributed asymmetrically is determined by the direction of critical slowing down. The application of a detrending method may prolong this period when trends are strong.

Qualitatively similar results were also found when analysing data sets of communities with different numbers of species (see Appendix S7). Full network collapses became less common as the number of species increased, and Hopf bifurcations leading to oscillatory, chaotic or other complex dynamics became more frequent (Fig. S21, S22). These changes occurred, most likely, due to a change in the balance between intra‐ and interspecific competition. Interaction strengths were assigned such that the relative difference between intra‐ and interpecific competitive interaction strengths remained approximately the same (see Appendix S5). The number of interspecific competitive interactions, however, increases as the number of species increases. The combined effect of all interspecific competitive interactions is therefore larger. Systems with many species may, due to the way in which we assigned competitive interaction strengths, therefore be comparable with smaller networks in which interspecific competition is relatively strong.

Simulations with the more general, unipartite model of facilitation between species gave roughly the same qualitative results as the bipartite plant‐pollinator model (see Appendix S7). The resilience of communities of 10, 20 and 40 species was generally a bit lower than the resilience of plant‐pollinator networks with the same number of plant and pollinator species. To prevent networks from collapsing almost immediately, we chose a lower noise level with standard deviation $\delta_{i}=0.05$. A relatively low resilience may also explain the relatively high frequency of likely cascading collapses in facilitative communities of 10 species (Fig. S23). A different way of assigning critical abundances, *A_i_*, could have increased the resilience of the here studied facilitative communities.

## DISCUSSION

Human activities alter the Earth's climate and its ecosystems at unprecedented rates (Vitousek *et al.* 1997; Millenium Ecosystem Assessment 2005; Rockström *et al.* 2009; IPCC 2014; Steffen *et al.* 2015). These changes may jumble the patterns in the networks of interactions between species that hold complex species communities together (Kareiva *et al.* 1993; McCann 2007; Tylianakis *et al.* 2008). Monitoring and forecasting the effects of such changes thus requires a systems approach; an approach that explicitly studies the properties emerging from the complex and often unknown ways in which species relate to each other. Here, we try to make a further step towards developing such an approach by determining the direction in which destabilising positive feedbacks undermine resilience. With model‐generated time series, we show that this direction is indicative of the future state of mutualistic communities, potentially providing us with a tool to assess the impact of impending critical transitions in natural communities and other complex systems.

Ecologists have emphasised the importance of improving our ability to predict the future state of ecosystems previously, and predicting future developments in complex systems is common practice in various fields of research, for example in economics, engineering, and climatology (Clark *et al.* 2001; Sutherland 2006; Coreau *et al.* 2009; Beckage *et al.* 2011; Novak *et al.* 2011; Evans *et al.* 2012, 2013; Purves *et al.* 2013; Petchey *et al.* 2015). Concerns about the forecastability of ecosystems and the limits to our capacity to predict the future state of ecosystems have, however, also been strong (Coreau *et al.* 2009; Beckage *et al.* 2011). Some of these concerns stem from a misunderstanding of why predictions are made. Making predictions is fundamentally different from describing a scientific law. Predictions are made when a limited amount of knowledge is available, and people rely on predictions, even when they are known to often be inaccurate, simply because better predictions are not available. Predictions may also be made when evaluating the risks associated with different ecological scenarios. In this spirit, we also see the indicator we propose here; as an indication of where a system's future state might lay. There is no absolute certainty as complex dynamics may occur after a critical threshold is passed.

Some general properties may, however, give a clue about the predictability of ecosystem dynamics. We found that, as the strength and variability of interspecific competition increases, dynamics change from a situation where positive feedbacks are the main cause of instability, to a mixed, intermediate situation, and, eventually, to a situation in which delayed negative feedbacks govern ecosystem dynamics. Our results suggest that the indicator performs well at predicting a system's future state when positive feedbacks are strong. Performance was reasonably good and transitions caused by positive feedbacks remained quite common in the aforementioned mixed situation (i.e. more than 50% accurate predictions). When dynamics were governed by delayed negative feedbacks, we found that the initial pristine state of the here studied systems was unlikely to be stable (i.e. the probability of a system's non‐trivial equilibrium to be stable was below 0.01). The indicator could not be applied and the interplay between several delayed negative feedbacks was likely to lead to chaotic dynamics.

Ecosystems exhibit positive feedbacks when species have direct or indirect positive effects on themselves (i.e. in loops with an even number of negative interactions), and do not only occur as the result of mutually beneficial interactions. Positive feedbacks may, for example, also occur when species positively affect themselves by suppressing other species (e.g. between a pair of competing species and in three‐species omnivore loops in food webs, see Van Nes & Scheffer 2004 and Neutel & Thorne 2014). Despite a long‐standing interest in the occurrence of complex ecosystem dynamics (May 1974; Hastings & Powell 1991; Huisman & Weissing 1999), no real classification of where and when to expect unpredictable, complex dynamics exists. As a first speculative proposal, we suggest that all the various types of mutualistic communities are likely to exhibit relatively strong positive feedbacks and predictable dynamics. Terrestrial foodwebs, where the top‐down effects of herbivory are relatively small (Cyr & Face 1993), may fall in the aforementioned mixed category, while aquatic food webs are more likely to exhibit chaotic dynamics (e.g. Benincà *et al.* 2008). Complex dynamics are likely to occur in competitive communities when competitive interaction strengths are variable and asymmetrical. When pairs of interacting species have similar competitive effects on each other, positive feedbacks between some pairs of species are more likely to be strong and dynamics may be fairly predictable (e.g. Van Nes & Scheffer 2004). Further research into where and when to expect complex dynamics will greatly improve our capacity to evaluate the performance of the here proposed indicator and the predictability of ecosystem dynamics in general. Such research may, for example, involve a further investigation of the interrelationship between the structural properties of ecological networks and the occurrence of different types of critical transitions and may include transitions that are not preceded by critical slowing down (see Grebogi *et al.* 1983 and Hastings & Wysham 2010).

Earlier studies explored different ways in which changing environmental conditions may lead to critical transitions in mutualistic networks, for example by increasing pollinator mortality rates (Lever *et al.* 2014; Jiang *et al* 2018) or by declining mutualistic interaction strengths (Dakos & Bascompte 2014). In this work, assumptions were made that make the effects of these changes fairly simple from a dynamical perspective (e.g. the assumption that the intrinsic properties of species and the effects of changing environmental conditions are similar for all species, and the assumption that the structure of whom interacts with whom remains unchanged). As a consequence, there is little change in the direction of slowest recovery and the nature of the systems' alternative stable states. Here, we chose to study a more complex dynamical scenario because we wanted to test whether the direction of critical slowing down is indicative of a community's future state even when the direction of slowest recovery changes substantially prior to the period in which resilience is lost. There is no reason to assume that the indicator would perform worse at predicting a system's future state when changing conditions affect a group of similar species in one of the aforementioned more simple ways.

The here proposed indicator has a number of advantages compared to previous methods to predict the future state of ecosystems such as extrapolation and the use of mechanistic models. Extrapolation is risky, because it assumes trends to continue outside of the range in conditions for which data are collected, and the behaviour of mechanistic models, for example aiming to simulate feeding, reproduction, death, and other rates with as much accuracy as possible, often depends on many unknown parameters, in particular when these rates depend on environmental conditions and species abundances. Using the direction of critical slowing down as an indicator of a system's future state has the advantage that it directly relates to an emerging property of complex ecosystems (i.e. the direction in which resilience is lost). As such, it avoids the often difficult process of parameter estimation needed to develop mechanistic models, and it specifically aims to predict a system's future state when abrupt shifts away from existing trends (i.e. critical transitions) occur.

The above‐described results consider scenarios in which plenty of data are available. When time series are short (i.e. contain few data points) or when the rolling window used to analyse time series contains few data points, predictions become less accurate (Fig. S24–S29). This brings us to the question of how we may determine the data requirements in practice. In this context, it is important to consider the two different aspects of our analysis: ‘critical slowing down’ and ‘the direction of slowest recovery’. Critical slowing down can only be detected over a longer time periods (i.e. in which conditions change) while the direction of slowest recovery can be determined for a given set of conditions (i.e. over a short period of time). When determining critical slowing down, it is not necessary to monitor the abundances of all species per se, while this is important when determining the direction of slowest recovery. A more economical approach could thus be to monitor only few species for indicators of critical slowing down, for example using the methods in Scheffer *et al.* (2009) and Dakos *et al.* (2012), and to determine the direction of slowest recovery only once these indicators suggest that the system approaches a tipping point. In some cases, one may even consider to skip monitoring of critical slowing down indicators altogether and focus on determining the direction of slowest recovery in systems that are known to be under stress.

Two aspects could cause our approach to be less data hungry than expected. First, we are only interested in the slope indicated by the first principal component and require, therefore, fewer data when compared to analysis in which also the higher‐order components are of importance. Second, we expect the distribution of abundances to become highly asymmetric when a system approaches a tipping point. Dynamics become similar to a low‐dimensional system and the number of observations needed to accurately determine the direction of slowest recovery becomes smaller when a system approaches a tipping point (Fig. S30). It remains, however, difficult to determine a priori what the data demands are.

Previous studies have proposed rules of thumb that give an indication of the minimum sample size required to perform principal component analysis (i.e. the method used to determine the slope of the indicator). Such rules are often a function of the number of variables (e.g. species abundances) and suggest that the minimum sample size required to perform a principal component analysis should be at least *n*, for example 2, 10 or 20, times more than the number of variables. Velicer & Fava (1998) and MacCallum *et al.* (1999) showed, however, that such rules of thumb are invalid and that the required sample size depends on the underlying correlation structure. A better approach to determine the minimum sample size is therefore to draw subsets from the data and compare results for the subset with those for the full set (Barrett & Kline 1981; Arrindell & Van der Ende 1985). When subsets give similar results to the full set, enough data is likely obtained. Methods to determine the effect of a change in sample size may vary from a simple comparison of the direction indicated (as in Fig. S30) to more advanced bootstrapping techniques (as in Shaukat *et al.* 2016).

In this study, we chose to use time‐series analysis because it links closely with previous work on early warning signals (Scheffer *et al.* 2009; Dakos *et al.* 2012), and because data collection efforts have, traditionally, focused on species abundances. For some ecosystems it may, however, be easier to monitor changes in the structural properties of ecological networks rather than in the specific way in which a system recovers from small perturbations. When such monitoring efforts could be used to estimate (changes in) the effective relationships between species as described by the different elements of the Jacobian matrix, we may be able to obtain a more direct measure of (changes in) the relative strengths of feedback loops in ecosystems, their proximity to a tipping point, and their likely future states. Our analysis suggests, for example, that the extent to which species are saturated and the relative benefits received from mutualistic partners play a crucial role in determining the resilience and future state of mutualistic communities. These properties might be measured in more direct ways, for example by determining the time spent by pollinators on handling and searching for nectar and their relative visitation rates to different plant species. Other theoretically informed measures for other types of ecosystems may likely provide us with other potential indicators of the direction of critical slowing down.

In a time when humanity's biggest challenges and opportunities depend upon our capacity to manage complex natural systems, new tools to foresee the risks and opportunities associated with critical transitions are of increasing importance. Such tools may not only be useful when addressing the question of what a system's future state might be like, but may also help to address questions such as to what extent individual species or interactions are contributing to network resilience and which deliberate human interventions could prevent or alter the outcome of impending critical transitions. Such approaches are becoming increasingly useful as the availability of data on natural and other complex systems is rapidly increasing.

## AUTHORSHIP

J.J.L., I.A.L., E.W., R.Q. and E.N developed the methods. J.J.L. performed the simulations and data analysis, and prepared the first draft of the paper. All authors contributed to the design of the research, discussed data and contributed to writing the final manuscript (in particular J.B. and M.S.).

## DATA ACCESSIBILITY STATEMENT

Code used to generate and analyse time series is available at https://github.com/JJelleLv/MutNetBeyondCollapse and https://doi.org/10.5281/zenodo.3458586.
