# Supplementary material for: Foreseeing the future of mutualistic communities beyond collapse
Source: Ecol Lett. 2019 Nov 10;23(1):2–15. doi: 10.1111/ele.13401 (PMC6916369; doi:10.1111/ele.13401)
Supplement: Supplementary file 1 [file ELE-23-2-s001.pdf]

Foreseeing the future of mutualistic communities beyond collapse

– Supporting Information –

J. Jelle Lever[1,2,*], Ingrid A. van de Leemput[2], Els Weinans[2], Rick Quax[3], Vasilis Dakos[4], Egbert H. van Nes[2], Jordi Bascompte[1], and Marten Scheffer[2]

[1]Department of Evolutionary Biology and Environmental Studies, University of Zurich, Winterthurerstrasse 190, CH-8057 Zurich, Switzerland.

[2]Department of Aquatic Ecology and Water Quality Management, Wageningen University, P.O. Box 47, NL-6700 AA, Wageningen, The Netherlands.

[3]Computational Science Lab, University of Amsterdam, NL-1098 XH, Amsterdam, The Netherlands

[4]Institut des Sciences de l'Evolution de Montpellier (ISEM), BioDICée team, CNRS, Université de Montpellier, Montpellier, France

*Correspondence: E-mail: jelle.lever@ieu.uzh.ch.

S1 EXAMPLE: UNDERMINING THE RESILIENCE OF A 3-SPECIES NETWORK

To illustrate how differences in the intrinsic properties of species and the arrangement of interactions between them may affect the overall resilience of mutualistic networks, we use a model in which one pollinator species interacts mutualistically with two plant species. The system's overall resilience is highest when this pollinator species obtains most resources from the more saturated plant species.

As conditions change from a situation in which pollinators obtain most resources from highly saturated plant species P_1, i.e. with high saturation term h_1, to a situation in which they obtain most resources from less saturated plant species P_2, the network becomes increasingly sensitive to small-scale stochastic perturbations. Eventually, a critical transition occurs away from the initial pristine state of the network towards a fully collapsed network state in which both plant species and the pollinator species are extinct.

For illustrative purposes, we assume plants to be in steady-state and determine how changing conditions affect the relationship between the net growth of the pollinator species, $dN^{(A)}/dt$, and the abundance of the pollinator species, $N^{(A)}$ (Fig. S1). The net growth of the pollinator species is negative at low abundances. As a result, there are two alternative stable states; a pristine state in which the pollinator species has a positive abundance and a collapsed state in which the abundance of the pollinator species is zero. These two alternative stable states can be visualized more intuitively by a stability landscape of which the slope corresponds to the rate at which the abundance of the pollinator species changes, $dN^{(A)}/dt$, valleys to the attraction basins of the alternative stable states, and hilltops to the threshold between the two attraction basins. As conditions change, the attraction basin of the initial pristine state of the network becomes increasingly small and a small perturbation becomes sufficient to cross the threshold and cause a critical transition towards the alternative fully collapsed state of the network.

Parameter settings: $\hat{N}_i = 2$, $c_{ii} = 0.4$, $c_{ij} = 0.1$, $d_i = 0.2$, $h^{(A)} = 0.3$, $h_1^{(P)} = 0.3$, $h_2^{(P)} = 0.1$, and $\epsilon_i = 0.01$. Initial interaction strengths: $(M = 0)$: $\theta^A_{0,11} = 1$, $\theta^A_{0,12} = 0$, $\theta^P_{0,11} = 1$, and $\theta^P_{0,21} = 1$. Final interaction strengths: $(M = 1)$: $\theta^A_{final,11} = 0$, $\theta^A_{final,12} = 1$, $\theta^P_{final,11} = 1$, and $\theta^P_{final,21} = 1$.

S2 EXAMPLE: CRITICAL SLOWING DOWN IN A 4-SPECIES NETWORK

To illustrate the direction in which a community slows down prior to a critical transition and how this might be used to predict a community's future state, we use a model in which two pollinator species interact mutualistically with two plant species. As described in the main text, changing conditions undermine the resilience of this small network by altering relative mutualistic benefits, θ. As was the case with the earlier studied 3-species network (see Appendix S1), regime shifts occur in the here studied 4-species community because the community's initial pristine state is approached by a threshold (i.e. a boundary between two attraction basins, Fig. 1). As conditions change, the minimum size needed for perturbations to push the system over the approaching threshold becomes smaller. The likelihood of a transition caused by the small-scale stochastic perturbations incorporated in our model therefore increases and, eventually, a regime shift towards an alternative state becomes inevitable.

The outcome of a transition depends on the way in which changing conditions undermine a community's resilience. One, some or all species may collapse to extinction and remaining species may either gain or lose in abundance from a regime shift. Multiple thresholds separating the community's initial pristine state from different alternative stable states, or 'potential future states', may exist prior to a regime shift. Changing conditions may alter the number and nature of these alternative stable states, and the thresholds towards them may or may not approach the network's initial pristine state. Which alternative state eventually becomes the community's future state depends on which threshold towards which future state eventually approaches a community's initial state.

For illustrative purposes, we assume plants to be in steady state and determine how changing conditions affect the dynamics of the network. These dynamics can be visualized intuitively by a stability landscape of which the slope corresponds approximately to the rate at which the abundances of pollinator species change, $dN^{(A)}/dt$ (see methods below). Every possible combination of pollinator abundances is represented by a unique point in the stability landscape and alternative stable states are at the lowest point of the landscapes valleys or 'attraction basins'. Thresholds between attraction basins are represented by ridges in the stability landscape. These thresholds are not equally high at all places and have local maxima at hilltops and local minima at saddle points in the network's stability landscape. Attraction basins are shallow in between alternative stable states and the saddle points on the thresholds that separate them. When approached by a threshold, the attraction basin of the initial pristine state becomes increasingly shallow and the network increasingly slow when recovering from perturbations in the direction of the saddle point on the approaching threshold.

For the here studied 4-species network (Fig. 1) we found that the network's pristine state is initially accompa-

nied only by a fully collapsed state, i.e. a state in which the abundance of all species is zero. The pristine state's distance from the threshold towards this state, however, remains large even when conditions change. A regime shift towards a fully collapsed state remains, therefore, unlikely. Changing conditions start to rapidly undermine the network's resilience only after the appearance of the first of two additional alternative stable states. These states correspond to partially collapsed network states in which the abundance of some but not all species is zero. Both thresholds towards both partially collapsed states approach the network's pristine state. One threshold, however, approaches the initial pristine state more closely than the other and eventually a regime shift, caused by the small-scale stochastic perturbations to which the network is permanently subjected, towards the partially collapsed state in the attraction basin behind this threshold becomes inevitable.

As conditions change there are two decisive moments which are both preceded by a particular change in the network's dynamics. The first is the moment at which the future state of the network comes into existence as an alternative stable state in the network's stability landscape, and the second is the moment at which the regime shift towards this alternative stable state actually occurs. The direction in which the network recovers slowly from perturbations changes substantially before the future state of the network comes into existence from a direction that roughly indicates a full collapse to a direction that indicates the future partially collapsed state of the network. The speed at which the network recovers from perturbations, however, remains approximately the same. After the future state of the network comes into existence, the network slows down dramatically when recovering from perturbations in approximately the same direction (Fig. S2).

Methods: To determine the rate at which pollinator abundances change as illustrated in Fig. 1.b, we analytically determined this rate, $v^{(A)}$, for different pollinator abundances at 200 by 200 grid points in the network's phase plane as follows:

$$v^{(A)} = \left(\sum_{i=1}^{S^{(A)}} \left(\frac{dN_i^{(A)}}{dt} \right)^2 \right)^{0.5}, \tag{S1}$$

in which $N_i^{(A)}$ is the abundance and $dN_i^{(A)}/dt$ the net growth rate of pollinator species i. At the same grid points we determined the height of the stability landscape with an algorithm that keeps updating the height of the landscape until all slopes in between these points are within a certain margin of error from the pollinators net growth rate. This allows us to intuitively show the position of alternative stable states, which are found at the bottom of the landscapes valleys or 'attraction basins', and the thresholds between them, which correspond to hills or ridges in the landscape. The stability landscape produced with this algorithm, is a useful tool to intuitively illustrate the idea behind our method. As our system is non-gradient, it is not a way to determine the potential energy of the system.

Parameter settings: $\hat{N}_i = 2$, $c_{ii} = 0.4$, $c_{ij} = 0.1$, $d_i = 0.2$, $h_1^{(A)} = 0.1$, $h_2^{(A)} = 0.3$, $h_i^{(P)} = 0.3$, and $\epsilon_i = 0.04$. Initial interaction strengths: $(M = 0)$: $\theta_{11}^A = 0.7$, $\theta_{12}^A = 0.3$, $\theta_{21}^A = 0.5$, $\theta_{22}^A = 0.5$, $\theta_{11}^P = 0.5$, $\theta_{12}^P = 0.5$, $\theta_{21}^P = 0.3$, $\theta_{22}^P = 0.7$. Final interaction strengths: $(M = 1)$: $\theta_{11}^A \approx 0.83$, $\theta_{12}^A \approx 0.17$, $\theta_{21}^A \approx 0.10$, $\theta_{22}^A \approx 0.90$, $\theta_{11}^P \approx 0.90$, $\theta_{12}^P \approx 0.10$, $\theta_{21}^P \approx 0.17$, and $\theta_{22}^P \approx 0.83$

Conditions analyzed for Fig. 1 in the main text: $M = 0.31$, $M = 0.66$, and $M = 0.87$.

S3 SIMILARITY BETWEEN THE INDICATED AND OBSERVED SHIFT

As explained in the main text, the slope of the indicator is determined by the first principal component (Fig. S4.C), while the eventual (up- or downward) direction of the indicator along the first principal component is determined by the direction in which time points are skewed (Fig. S4.D-E). To asses the performance of our indicator, we evaluate the performance of the first principal component and the skewness of the projected time points independently. An accurate slope, means that the indicator performs well at predicting the relative gain or loss of species and which species shift in opposite directions (i.e. an 'accurate PC1'). The indicated direction is, however, only fully 'accurate' when the actual winners and losers are also indicated correctly. This depends on the direction along the first principal component in which time points are skewed.

To evaluate the performance of the first principal component, we determine the difference between the slope of our indicator and the direction of the observed shift in abundance. We do this by determining the angle, θ, between the direction of the indicator and the observed shift as follows:

$$\theta = \cos^{-1} \frac{I \cdot \Delta N^{(A)}}{|I||\Delta N^{(A)}|}, \tag{S2}$$

in which I is the indicator of a network's future state and $\Delta N^{(A)}$ the observed shift in pollinator abundances. $I \cdot \Delta N^{(A)}$ indicates that we take the dot product between these two vectors. To determine $\Delta N^{(A)}$, we take the mean abundances over 200 time steps at 500 steps before the tipping point and subtract it from the mean abundances 500 steps after the tipping point was found. Because we want to evaluate the accuracy of the first principal component, and not whether points are also skewed in the right direction, we take $-I$ as the input for the formula above when we find an angle $> \pi/2$ (i.e. $>$ 90 degrees). Both I and $\Delta N^{(A)}$ are vectors of which the number of dimensions is equal to the number of species analyzed. The smaller the angle, the more similar the direction of the two vectors.

Two random vectors in a ten-dimensional space are more likely to be orthogonal than two random vectors in a three-dimensional space. More extreme small or large angles become less likely as the number of dimensions increases (Fig. S5). How 'special' it is to find a certain angle between the indicated and the observed shift thus depends on the number of dimensions in a system. As a measure of how different the indicated direction is from the observed regime shift, we determine for the observed angle, θ, the likelihood that two unrelated random vectors have an equal or smaller angle. As a measure of similarity, we take one minus this probability, and we consider the indicator's slope to be accurate when this measure of similarity is above 0.99.

To determine the aforementioned probability, we use the following probability density function:

$$h(\theta) = \frac{1}{\sqrt{\pi}} \frac{\Gamma(\frac{S^{(A)}}{2})}{\Gamma(\frac{S^{(A)}-1}{2})} \cdot (\sin\theta)^{S^{(A)}-2}, \tag{S3}$$

in which $S^{(A)}$ is the number of dimensions and $h(\theta)$ the probability density for a certain angle θ (ref. Cai *et al.* (2013)). Our method may be interpreted as a test whether the null hypothesis that I and N are two random vectors is true. This hypothesis is rejected when angle is found to be significantly smaller than the expected angle between two random vectors, when the one-sided p-value is smaller than 0.01 (i.e. similarity $>$ 0.99).

To evaluate the tendency of time points to be skewed in the direction of a network's future state, we determine the skewness of the time points projected on the first principal component. When points are skewed in the direction of the network's future state, we report a positive skewness. When points are skewed in the opposite direction, we report a negative skewness. We consider a positive skewness as accurate and a negative skewness as inaccurate. A strong positive or negative skewness is considered more accurate or inaccurate than a weak positive or negative skewness.

S4 TIME SERIES ANALYSIS

Unless stated otherwise, we determine the dominant direction of fluctuations in a rolling window of 10% of the entire time series (e.g., 2000 out of 20.000 time points) to detect changes in the direction and extent in which time points are distributed asymmetrically. The choice of this window size is to some extent arbitrary. A too small window leads to irregular trends, while a too large window smooths out the trends. To test whether the size of the window chosen influences our results, we make additional analysis in which we use a window size of 0.005, 0.1, 0.5, 0.1, 5,10, 20 and of 50% of the time series. The rolling window is moved along the time series with steps of 1% of the time series, independent of the window size. As time passes by, the direction and magnitude of the indicator is thus computed every 200 time steps in a window containing the last 2000 time steps when using a window size of 10% of a time series with a length of 20.000 time points.

Far from a tipping point, time points may be skewed only weakly. When this is the case, sudden shifts of nearly 180 degrees may occur in the direction of the indicator when time points are skewed in a different direction along the first principal component. Clearly, such large shifts in direction do not occur because the network's future state has changed. We, therefore, correct previously found indicator values such that there is no change larger than 90 degrees between two consecutive points at which the indicator's direction was determined. We assume the last direction in which time points were found to be skewed to be the accurate one.

To determine whether there is a significant increase in the indicator's magnitude, we determine the Kendall rank correlation coefficient, τ, for the last ten points at which the indicator's magnitude was computed. We consider the increase significant when this coefficient was positive and its p-value < 0.05. Once a significant increase was found, we tested whether the increase remained significant by determining Kendall's correlation for the last eleven points the next time the indicator's magnitude is determined, for twelve points the time after that, and so on until the tipping point is reached. We would again look at the last ten points when the increase was found to not be significant anymore. By doing this, we could determine the range in conditions in which the indicator's magnitude increased significantly.

As a measure of a 'regime shift' we determined whether there was a change in abundance of more than 1.5 over a period of 1% of the entire time series (200 time steps). We did this by taking the mean abundances over a period of 200 time steps before this period and 200 time steps after this period and determining Euclidean distance between these two mean abundances. To make sure that this large shift in abundances was not a temporal large deviation from the species' mean abundances, we added as a second criterion that the abundance of at least one

species should be near extinction, i.e. below 0.1.

We did not apply any preprocessing to handle trends in the time series. We expect the indicator to be relatively robust against such trends, because trends only alter the direction of the first principal component when their effect on this direction is stronger than the effect of critical slowing down. Not applying any preprocessing is a good way to test this robustness. When using the indicator as part of a different study it may, however, be worth considering to apply a preprocessing method (see ref. Dakos *et al.* (2012)). It may improve the performance of the indicator, especially when trends are strong.

S5 ADDITIONAL INFORMATION BIPARTITE MUTUALISTIC NETWORKS

Nontrivial equilibrium abundances, $\hat{N}$, competitive interaction strengths, c, mortality rates, d, and saturation terms, h, are randomly sampled from predefined probability distributions, and the total amount of resources received by species i at the system's nontrivial equilibrium, $R_i(\hat{N}^{(P)})$, are assigned such that the rate at which abundances change at the system's nontrivial equilibrium, $d\hat{N}^{(P)}/dt$, is zero:

$$R_i(\hat{N}^{(P)}) = \frac{\sum_{j=1}^{S^{(A)}} c_{ij}\hat{N}_j^{(A)} + d_i}{1 - h_i(\sum_{j=1}^{S^{(A)}} c_{ij}\hat{N}_j^{(A)} + d_i)}. \tag{S4}$$

The total amount of resources provided at the system's nontrivial equilibrium, $R_i(\hat{N}^{(P)})$, is thus approximately the same for highly specialized and more generalist species, provided that their losses due to competition, c, and mortality rates, d, and their nontrivial equilibrium abundances, $\hat{N}$, are similar.

The extent to which species are saturated is determined by the total amount of resources provided, $R_i(\hat{N}^{(P)})$, and the rate at which species become saturated as determined by saturation term h_i. In our simulations, we assume nontrivial equilibrium abundances, $\hat{N}$, and inter- and intraspecific competition, c_{ij} and c_{ii}, to be similar for all species. Highly saturated species are, therefore, the ones with a high h_i. Species are saturated relatively quickly, and, according to equation S4, the total amount of resources provided at the system's nontrivial equilibrium is high when species have a high h_i.

Parameters are assigned such that there are substantial differences in the extend in which species are saturated by drawing saturation terms, h_i, from a scaled beta distribution with range $\sim (0.05, 0.35)$ and shape parameters $\alpha = 1$ and $\beta = 5$. Due to this distribution, there are few highly saturated species, i.e. h_i close to 0.35, and many non-saturated species, i.e. h_i close to 0.05. Strong mutualistic interactions between non-saturated species lead to strong positive feedbacks. Non-saturated species thus need to obtain a relatively large share of resources from a few, highly saturated species for the network to be stable. Relative mutualistic benefits at initial conditions, $\theta_{0,ik}$, are therefore ordered such that larger benefits are obtained from the more saturated species. To make sure that the sum of all relative benefits is one, we take relative mutualistic benefits, $\theta_{0,ik}$, from a symmetric Dirichlet distribution. The distribution's concentration parameter, α, determines the extent in which species are specialized and is, for each species, taken from a uniform distribution between zero and one.

To explore how transitions towards oscillating, chaotic or other complex dynamics caused by delayed negative feedbacks may influence the performance of the indicator, we analyze several data sets of which the strength and

variability in interspecific competitive interaction strengths, c_{ij}, varies. The tested parameter ranges are: $c_{ij} = 0$, $c_{ij} \sim U(0.02, 0.08)$, $c_{ij} \sim U(0.04, 0.16)$, $c_{ij} \sim U(0.06, 0.24)$, $c_{ij} \sim U(0.08, 0.32)$, $c_{ij} \sim U(0.10, 0.40)$, $c_{ij} \sim U(0.12, 0.48)$, and $c_{ij} \sim U(0.14, 0.56)$. Intraspecific competition strengths, c_{ii}, are taken from $\sim U(0.9, 1.1)$. Delayed negative feedbacks become stronger as the strength and variability of interspecific competition increases. Simulations are made for communities of 10 plant and 10 pollinator species. Initial equilibrium abundances, $\hat{N}_{0,i}$, and mortality rates, d_i, are taken from $\hat{N}_{0,i} \sim U(1.5, 2.5)$ and $d_i \sim U(0.15, 0.25)$. Initial and final nontrivial equilibrium abundances are assumed to be equal, $\hat{N}_{final,i} = \hat{N}_{0,i}$.

Changing environmental conditions, M, lead to an increase in the relative mutualistic benefits received from some, and a decrease in the relative benefits received from other species. We assume the distribution of interaction strengths of the final network, at $M = 1$, to be quite heterogeneous (Fig. S6). We select, therefore, with a probability of 0.75, interactions of which the interaction strength goes to zero, $\theta_{final,ik} = 0$. To the remaining interactions, relative interaction strengths are assigned by taking them from a uniform Dirichlet distribution ($\alpha = 1$). The 'diet breath' of plants and pollinators thus tends to become more narrow as could be the case under various scenarios of global environmental change (Memmott *et al.* 2007; Burkle *et al.* 2013).

As conditions change, either a single eigenvalue or a pair of complex conjugate eigenvalues goes to zero. In the first case we are dealing with a saddle-point approaching the network's initial state, caused by a positive feedback. In the second case, we are dealing with a Hopf bifurcation caused by a delayed negative feedback.

Data sets consist of 100 initial networks. For each network, 10 final distributions of relative mutualistic benefits, $\theta_{final,ik}$, were drawn, allowing us to determine the extent in which a community's future state depends on the specific way in which relative mutualistic benefits are changed. Parameters were assigned such that this dependency is high. Networks were discarded from a data set when they were unstable at initial conditions, $M = 0$. We determined the frequency at which this occurred as a measure of how difficult it is to find a stable solution for the initial networks of a given data set. The final distribution of relative mutualistic benefits was redrawn either when the network would become unstable within the range of conditions $M = (0, 0.5)$, or when a network would still be stable at $M = 1$.

To test whether the indicator also works when equilibrium abundances change, we analyzed networks of 10 plant and 10 pollinator species of which the final equilibrium abundances are different. We do this by changing the nontrivial equilibrium abundances of species as follows:

$$\hat{N}_i^* = \hat{N}_{0,i} + (\hat{N}_{final,i} - \hat{N}_{0,i})M, \tag{S5}$$

in which $\hat{N}_{0,i}$ is the initial, $\hat{N}_{final,i}$ the final, and $\hat{N}_i^*$ the actual nontrivial equilibrium abundance of species i. The total amount of resources provided at the system's nontrivial equilibrium, and the strengths of mutualistic interactions are determined by equations 4 and S4. We tested three scenarios. One in which the nontrivial equilibrium abundances of species tend to increase, $\hat{N}_{final,i} \sim U(2,3)$, one in which they stay the same on average $\hat{N}_{final,i} \sim U(1.5,2.5)$, and one in which they tend to decrease $\hat{N}_{final,i} \sim U(1,2)$. Competitive interaction strengths were taken from the following distributions: $c_{ii} \sim U(0.9,1.1)$ and $c_{ij} \sim U(0.02,0.08)$. Changing abundances affect all relationships as described by the Jacobian matrix. The main effect of a decline in abundance is, however, a reduction of the direct negative effects of species on themselves which undermines resilience. Increasing abundances tend to promote resilience.

To test whether the indicator may accurately indicate the future state of larger networks, we analyzed networks of 10 and 20, 10 and 40, 20 and 10, 20 and 20, 20 and 40, 40 and 10, 40 and 20, and 40 and 40 plant and pollinator species. We assigned competitive interaction strengths such that the rate at which species lose in abundance due to competition, $\sum_{j=1}^{S^{(A)}} c_{ij} N_j^{(A)} N_i^{(A)}$, is approximately the same for different numbers of species, as well as the relative difference between intra- and interpecific competition, c_{ij}/c_{ii}. When a species group consisted of 10 species we assumed $c_{ii} \sim U(0.9,1.1)$ and $c_{ij} \sim U(0.02,0.08)$. When a group consisted of 20 species $c_{ii} \sim U(0.67,0.82)$ and $c_{ij} \sim U(0.015,0.06)$, and when a group consisted of 40 species $c_{ii} \sim U(0.44,0.54)$ and $c_{ij} \sim U(0.01,0.039)$. Initial and final equilibrium abundances were assumed to be equal, $\hat{N}_{final,i} = \hat{N}_{0,i}$.

The amount of noise, determined by standard deviation δ, is assumed to be equal for all species. Unless stated otherwise, we assume standard deviation $\delta = 0.1$. Additional simulations were made with lower and higher noise levels, $\delta = 0.01$, $\delta = 0.05$, $\delta = 0.15$, and $\delta = 0.2$ to make sure that this does not qualitatively alter the results. Higher noise levels were not tested because they would lead to an almost immediate collapse. Unless stated otherwise, model generated time series had a length, T, of 20.000 time steps. Additional simulations were made in which time series had a length of 100, 200, 1.000, 2.000, 10.000, and 100.000.

S6 ADDITIONAL INFORMATION UNIPARTITE MODEL OF FACILITATION

Nontrivial equilibrium abundances, $\hat{N}$, interspecific facilitation rates, γ_{ij}, critical abundances A_i, interspecific competitive interaction strengths, c_{ij}, carrying capacities, K, and mortality rates, d, are randomly sampled from predefined probability distributions. Intraspecific facilitation rates, γ_{ii}, and intraspecific competition rates, c_{ii}, are one. To make sure that the rate at which abundances change at the nontrivial equilibrium, $d\hat{N}_i/dt$, is zero, we assign the intrinsic growth rates, r, as follows:

$$r_i = \frac{d_i \hat{N}_i A_i K_i}{(\sum_{j=1}^{S} \gamma_{ij} \hat{N}_j - A_i)(K_i - \sum_{j=1}^{S} c_{ij} \hat{N}_j) \hat{N}_i}. \tag{S6}$$

The contribution of species to the overall resilience of a network is determined by critical abundance A_i. Species with a high critical abundance, A_i, collapse more easily and the overall resilience of the community is highest when such species are facilitated by species with a low critical abundance. A change from such a distribution to a more random distribution of facilitative interaction strengths will undermine resilience. To generate time series in which the resilience of the here described facilitative communities is undermined, we assume that conditions, M, affect facilitative interactions as follows:

$$\gamma^*_{ij} = \gamma_{0,ij} + (\gamma_{final,ij} - \gamma_{0,ij})M, \tag{S7}$$

in which $\gamma_{0,ik}$ is the initial, $\gamma_{final,ik}$ the final, and γ^*_{ij} the actual facilitative interaction strength. Conditions, M, change from zero to one over time. We assume that the total amount of facilitation received, $\sum_{j=1}^{S} \gamma_{ij} Nj$, remains equal as conditions change. We therefore determine the final facilitative interaction strength as follows:

$$\gamma_{final,ij} = \frac{\theta_{ij} \sum_{k=1}^{S} \gamma_{ik} \hat{N}_k}{\hat{N}_j}, \tag{S8}$$

in which θ_{ij} is the fraction of the total facilitation received by species i from species j.

We assign parameters such that there are substantial differences in the critical abundances of species by drawing critical abundances, A_i, from a scaled beta distribution with $\alpha = 5$ and $\beta = 1$ and range $\sim (0, 1.5)$. Due to the beta distribution, there are few highly vigorous species (i.e. A_i close to 0) and many non-vigorous species (i.e. A_i close to 1.5). The initial facilitative interaction strengths are taken from the following uniform distribution: $\gamma_{0,ij} \sim U(0.2, 1.8)$. Initial facilitative interaction strengths are ordered such that species receive most facilitation,

i.e. highest $\gamma_{0,ij}$, from species with the lowest A_i. We assume that as conditions change, the strength of some facilitative interactions increases strongly while others approach zero. Final relative facilitative benefits, $\theta_{final,ik}$, are therefore selected with a probability of 0.75 and set to zero. To the remaining interactions, relative benefits are assigned by taking them from a uniform Dirichlet distribution ($\alpha = 1$). As with the model of mutualistically interacting species, we chose for this distribution of critical abundances, A_i, and facilitative interaction strengths γ_{ij}, because it leads to a high variety in potential future states to which a network may shift. Other parameters and equilibrium abundances are taken from the following uniform distributions: $\hat{N}_i \sim U(1.5, 2.5)$, $c_{ij} \sim U(0.04, 0.16)$, $d_i \sim U(0.15, 0.25)$.

Simulations were made with networks of 10, 20 and 40 species. As for the bipartite model of mutualistically interacting species, we assign parameters such that the rate at which abundance is lost due to competition, $\sum_{j=1}^{S} c_{ij}N_j/K_i$, remains approximately the same for different species numbers, as well as the relative difference between intra- and interpecific competition (see main text). Carrying capacities, K_i, were therefore taken from respectively $K_i \sim U(5, 6)$, $K_i \sim U(7.63, 9.15)$, and $K_i \sim U(12.89, 15.47)$, depending on the number of species.

The amount of noise, determined by standard deviation δ, is assumed to be equal for all species. For the results shown in this document we assume standard deviation $\delta = 0.05$. As with the model of mutualistically interacting species time series had a length, T, of 20.000 time steps.

S7 SUPPLEMENTARY RESULTS

Independent of the parameter ranges chosen, we found that regime shifts were preceded by a substantial period in which the indicator's magnitude increases significantly, i.e. the 'critical range'. Our indicator would, provided that the future state is indicated accurately, point towards a network's future state during a substantial part of this period (Fig. S7). In Fig. S8 we provide information about the critical ranges as observed in a single data set ($c_{ij} \sim U(0.02, 0.08)$). These results are exemplary for the other data sets and show that our indicator consistently indicates a network's future state during the period in which the network slows down.

Cascading collapses occur at an intermediate range of competitive interaction strengths most likely due to the nature of effective relationships between species, i.e. the combined effect of all direct and indirect interactions (Fig. S9). When there is no competition, effective relationships are positive and species collapse as one group. When competition is strong, most effective relationships are negative and species collapse independently. Cascading collapses are only likely when effective relationships are a mix of positive and negative relationships. When interspecific competitive interaction strengths, c_{ij}, were taken from $\sim U(0.02, 0.08)$, we found that such likely cascading, full network collapses took up a bit more than 12% of the data set. For specific parameter ranges not tested by us, this percentage may be higher.

In Fig. S10 we provide examples of two cascading collapses and one immediate network collapse. Species that collapsed a bit later, were also the ones for which the indicated loss in abundance was smallest, suggesting that the indicator indicates the initial regime shift accurately. The amount of time in between two consecutive partial network collapses can be extremely small. Also when cascades are not clearly visible, we suspect therefore that the inaccurate prediction of a full network collapse is caused by the occurrence of a cascading collapse.

In Fig. S13 we provide an example of a network for which the future state is hard to predict because it may shift to several alternative future states. When making five simulations in which relative mutualistic benefits, θ_{ik}, are changed in the exact same way by changing conditions, M, we found that the network shifted to four different future states. The future state of this network is determined by the only stochastic element in our model; the small-scale perturbations to which the network is permanently subjected. Our indicator accurately indicates two of the future states to which the network may shift, but does not indicate the other future states. A likely explanation for the several future states to which this system may shift is the fact that this system is approaching a Hopf bifurcation, leading to oscillating (Fig. S14), chaotic or other complex dynamics (Fig. S15). Such dynamics may explain a high sensitivity to perturbations in more than one direction.

In Fig. S18-S20, we show examples of time series in which not only the relative benefits, θ_{ij}, change over time. The nontrivial equilibrium abundances, $\hat{N}_i$, and thus the total gain from mutualistic interactions, $R_i(\hat{N}_i)$, changes as well. We found that a change in abundance over time does not have a strong effect on the performance of the indicator (Fig. S17). In comparison to data sets in which abundances stay (on average) the same, full network collapses are much less frequent when abundances increase and much more frequent when abundances decrease. Quite a large fraction of full network collapses is indicated accurately when abundances decrease. Cascading collapses may occur less frequently because all species experience a similar loss in resilience as a consequence of a decline in abundance. Another difference is that the length of the critical range tends to be a bit shorter when abundances in- or decrease.

In Fig. S21 and S22, we show that the indicator performs well, also when we apply our method to networks with different numbers of plant and pollinator species. Full network collapses become less common as the number of species increases, as well as the occurrence of cascading network collapses. An explanation for this effect of an increase in species number is that the loss in abundance due to competition with other species, $\sum_{j=1}^{S^{(A)}} c_{ij} N_j^{(A)} N_i^{(A)} - c_{ii} N_i^{(A)} N_i^{(A)}$, increases substantially as the number of species increases. Systems with many species may, therefore, be comparable with smaller networks in which interspecific competition is relatively strong. In those networks we also observed that full network collapses were less frequent. Increasing numbers of species did not have clear effect on the length of the critical range, nor on the fraction of the critical range in which the future state was indicated accurately by the slope of the indicator (Fig. S22). We did, however, found some effect on the skewness of time points projected on the first principal component. The frequency at which we found that points were skewed in the wrong direction increased as the number of species increases.

In Fig. S23, we show results for a more general model of competition and facilitation (see main text). The general behavior and performance of the indicator is similar to the results obtained with the mutualistic network model. The overall resilience of the networks tested seems a bit lower than the resilience of the mutualistic networks (this depends on parameter settings). To prevent networks from collapsing almost immediately, at $M \approx 0$, we chose a lower noise level of $\delta = 0.05$. This relatively low resilience may also explain the relatively high frequency of cascading collapses in networks of 10 species.

REFERENCES

Burkle, L. A., Marlin, J. C. & Knight, T. M. (2013). Plant-pollinator interactions over 120 years: loss of species, co-occurrence, and function. *Science*, 339, 6127, 1611–1615.

Cai, T., Fan, J. & Jiang, T. (2013). Distributions of angles in random packing on spheres. *J. Mach. Learn. Res.*, 14, 1, 1837–1864.

Dakos, V., Carpenter, S. R., Brock, W. A., Ellison, A. M., Guttal, V., Ives, A. R. *et al.* (2012). Methods for detecting early warnings of critical transitions in time series illustrated using simulated ecological data. *PLoS One*, 7, 7, e41010.

Memmott, J., Craze, P. G., Waser, N. M. & Price, M. V. (2007). Global warming and the disruption of plant–pollinator interactions. *Ecol. Lett.*, 10, 8, 710–717.

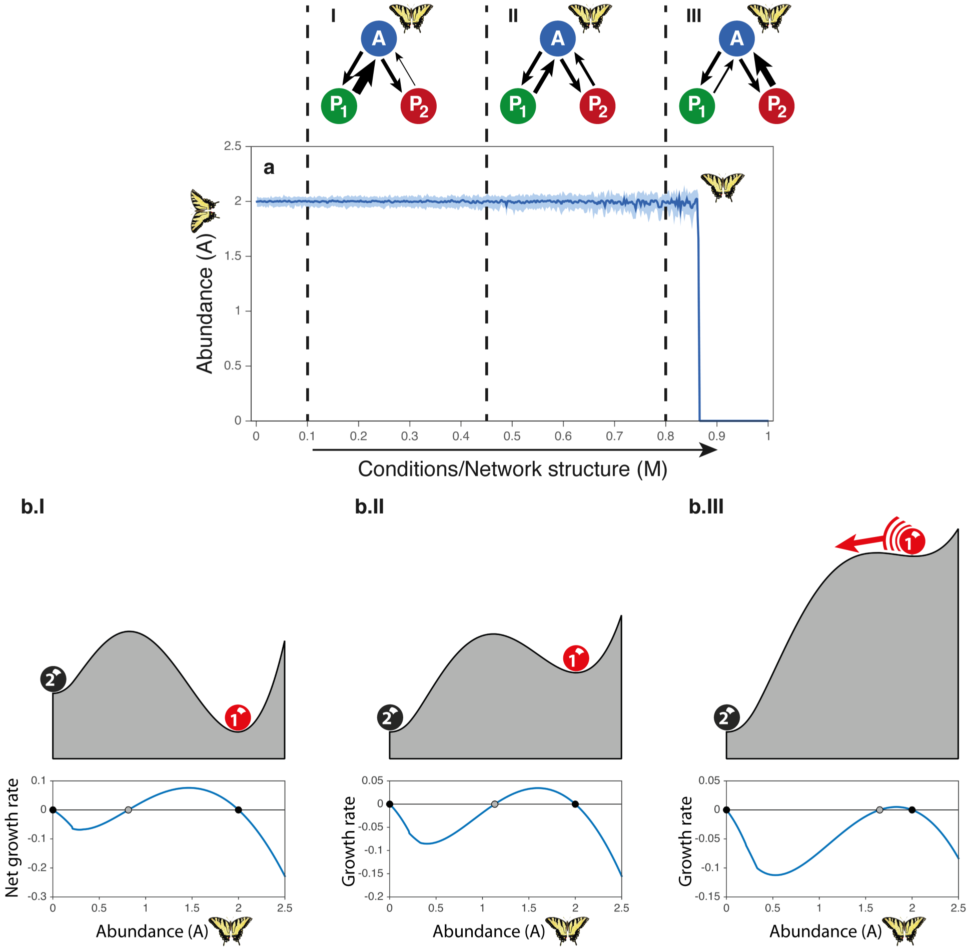

Figure S1 Changing conditions undermining the overall resilience of a small mutualistic network. The network consists out of one pollinator, A, and two plants species of which plant species P_1 is more saturated than plant species P_2. For illustrative purposes, we assume plants to be in steady-state. **(a)** Time series of the pollinator species and the network at different conditions (I, II, and III). As indicated by the thickness of the network's arrows, changing conditions alter the relative mutualistic benefits, θ, such that the pollinator species becomes increasingly dependent on non-saturated plant species P_2. This undermines the overall resilience of the network and leads to a full collapse of the network at which both plant species (not shown) and the pollinator species (shown) collapse to zero. **(b)** The net growth rate, dA/dt, and the stability landscape of the pollinator species at conditions I, II and III. As conditions change, the initial pristine state of the network, 1, is approached by a threshold, i.e. a hilltop in the stability landscape, and a small perturbation becomes sufficient to cause a regime shift towards fully collapsed state 2.

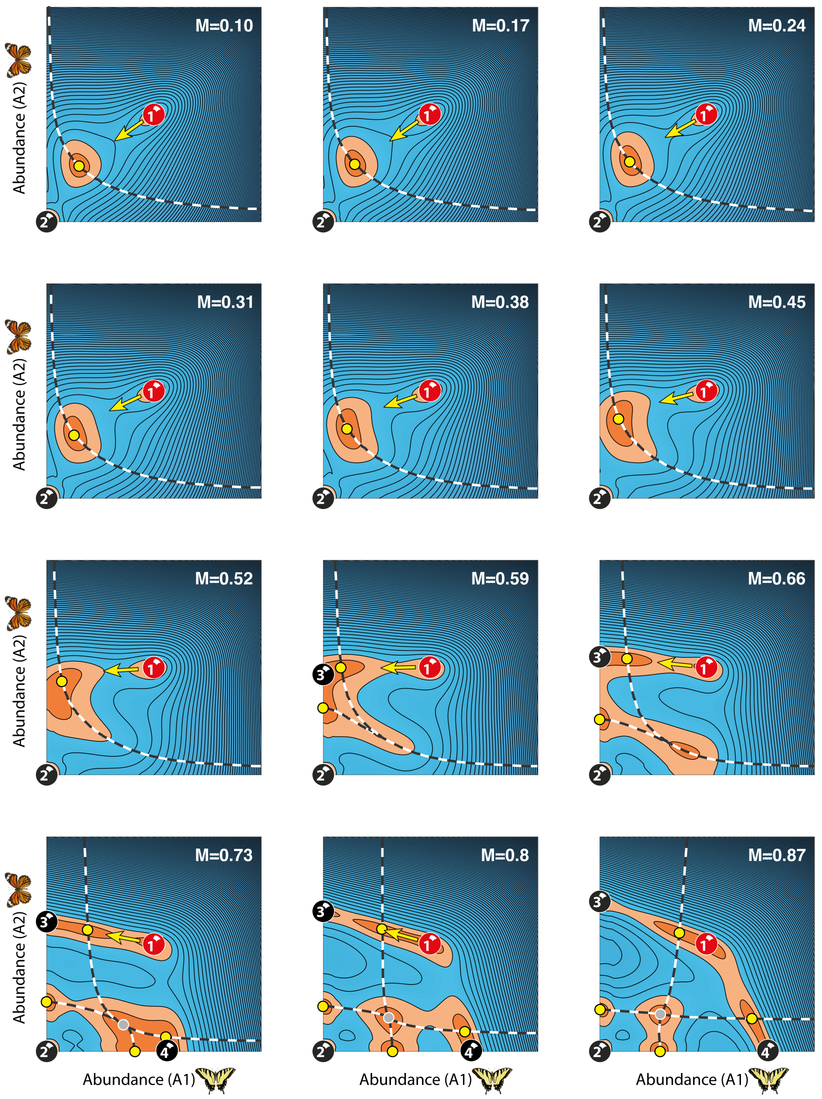

Figure S2 The slope of the small mutualistic network's stability landscape reflecting the speed at which pollinator abundances change, v_a, at different conditions, M. As in Fig. 2 of the main text, alternative stable states (balls), saddle points (yellow dots), and hilltops (grey dots) are surrounded by areas in which the landscapes slope, and thus the rate at which abundances change, is nearly zero (indicated in orange). Higher speeds (blue) are found further away from these points. The network recovers slowest from perturbations in the direction of the saddle point on the nearest threshold and slows down in the direction of the saddle point on the threshold approaching the network's initial pristine state. Changing conditions alter the shape of the network's stability landscape in a non-linear way. After a period in which there is almost no change ($M = [0, 0.31]$), the direction in which the network recovers slowest from perturbations (see yellow arrow) changes substantially from a direction that roughly indicates a full collapse to a direction indicating the future partially collapsed state of the network ($M = [0.31, 0.59]$). After the network's future state comes into existence, the network slows down dramatically in approximately the same direction ($M = [0.59, 0.87]$).

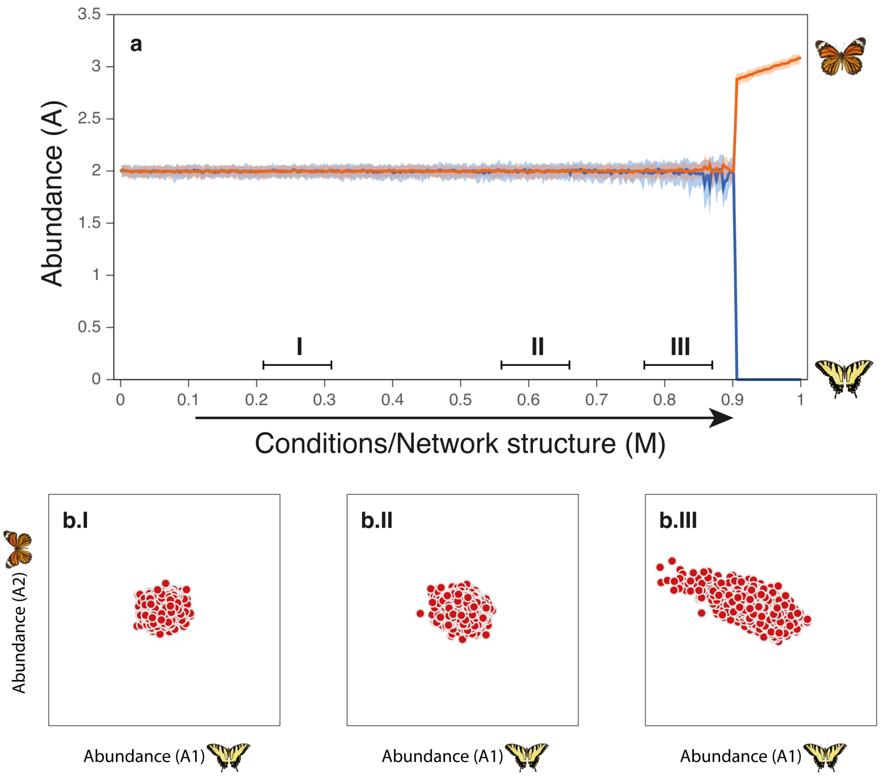

Figure S3 Example of a time series in which the small mutualistic network in Appendix S2 approaches a tipping point. Conditions at window I,II and III correspond to the conditions for which stability landscapes are shown in Fig. 1 of the main text. **(a)** At the tipping point ($M \approx 0.9$) one pollinator species collapses to extinction, while the other gains in abundance. **(b)** The distribution of points in the networks phase plane representing the abundances of species at different moments in time for time window I, II and III (see A). Far from the tipping point, in window I and II, deviations from the species' mean abundances are relatively small. Close to the tipping point, in window III, the distribution of points in the network's phase space is highly asymmetrical. Deviations from the mean abundances in time window III usually involve a simultaneous increase in the abundance of species A1 and a relatively larger decrease in the abundance of species A2, suggesting that this will also be the direction in which the network will shift once a threshold is passed.

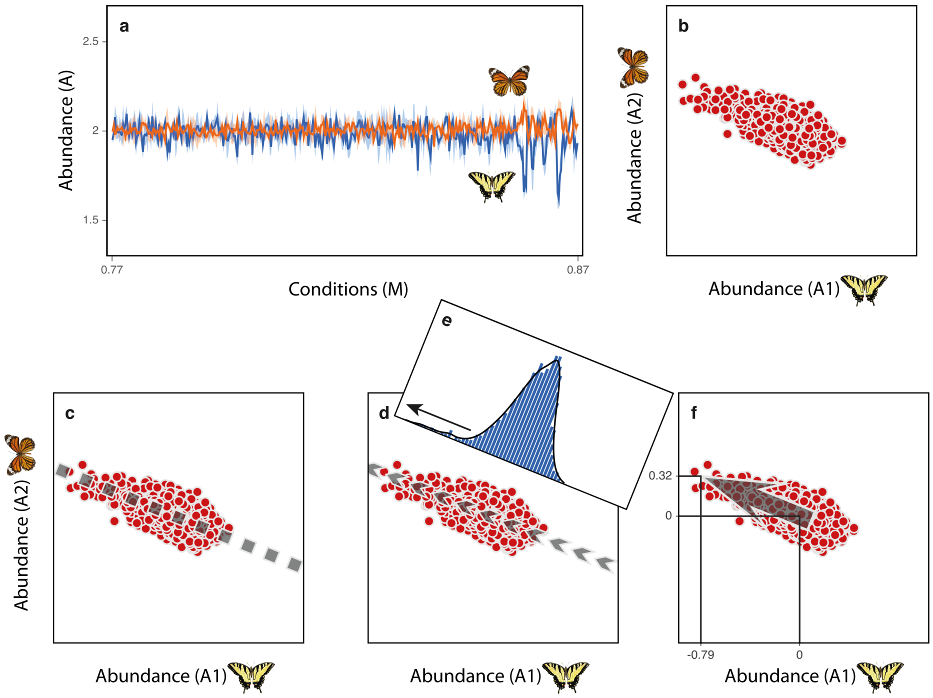

Figure S4 The measures of asymmetry together forming our indicator as they were determined for window III in Fig. S3. **(a)** Time series of the two pollinator species in the moving window. **(b)** Time points, representing species abundances at different moments in time, in the phase plane of the network. **(c)** The first principal component (grey dotted line) corresponding to the line in the phase plane along which variance is highest. **(d)** Direction along the first principal component (grey arrows) in which time points deviate the most from the species' mean abundance, i.e. the direction in which time points projected on the first principal component are skewed. **(e)** Distribution of the projected time points. **(f)** The indicator, corresponding to a vector in the phase plane of the network (grey arrow). The two components of this vector correspond to the species 'scores on the indicator'. In this example, we found a large negative score (-0.79) indicating a relatively large decline in abundance for the pollinator on the x-axis and a relatively smaller positive score (0.32) indicating a relatively smaller increase in abundance for the pollinator on the y-axis. The length of the indicator corresponds to the amount of variance explained by the first principal component.

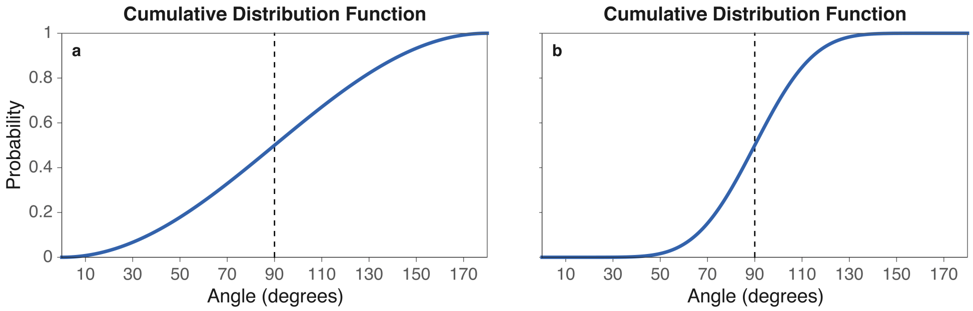

Figure S5 Cumulative distribution function of the angle between two random vectors. **(a)** Cumulative distribution function when these vectors have three dimensions. **(b)** Cumulative distribution function when these vectors have ten dimensions. As can be seen from the distributions, the probability of finding an angle of, for example, 40 degrees or less is much smaller in a high dimensional system. Cumulative distribution functions are determined with the help of the probability density function in ref. Cai *et al.* (2013).

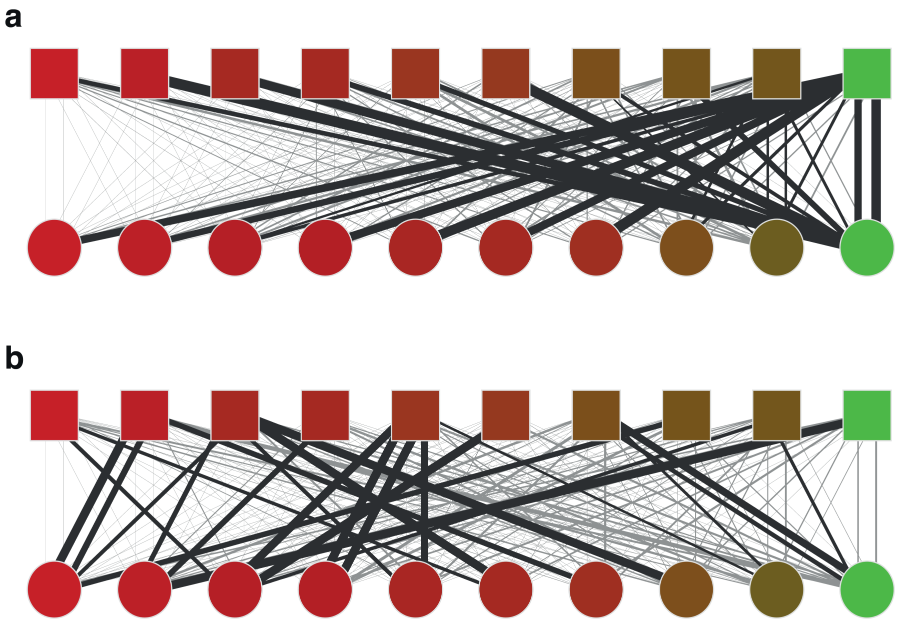

Figure S6 Example of **(a)** a highly resilient mutualistic network and **(b)** a network with a low resilience. Plant (circles) and pollinator species (squares) are ordered from highly saturated (green/left) to non-saturated (red/right). The thickness of the lines between nodes indicates relative mutualistic benefit θ_{ij}. In the highly resilient network species receive most of their resources from highly saturated species, while this is not the case in the network with a low overall resilience. The resilience of a network is undermined when relative benefits are changed from the situation in **a** to the situation in **b**.

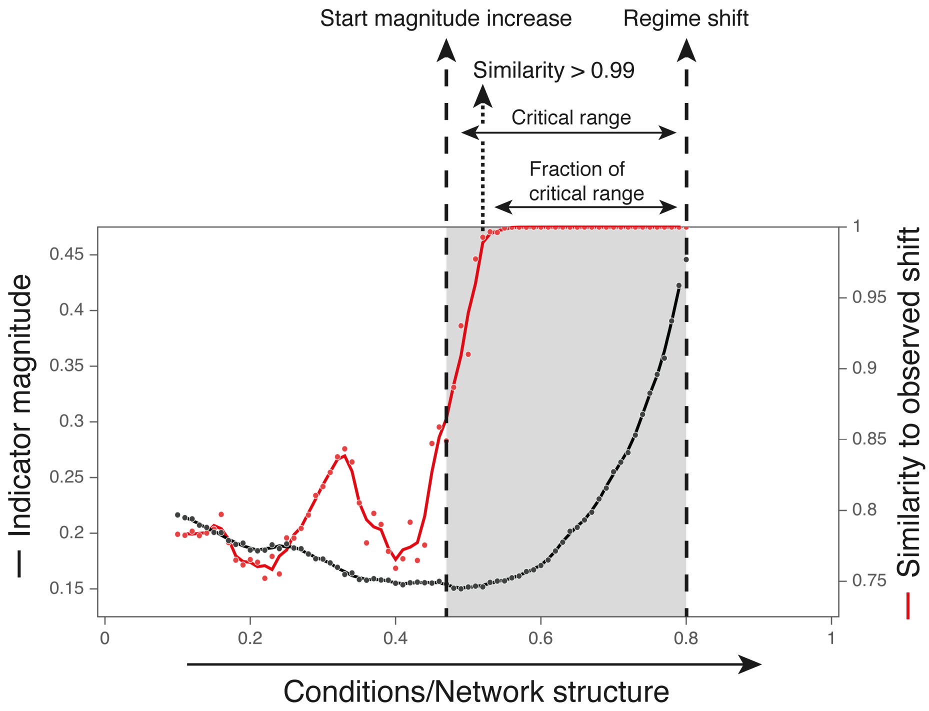

Figure S7 The critical range (grey band) in which the indicator's magnitude increases significantly and the fraction of this range in which the indicator's similarity to the observed shift in abundance is larger than 0.99. In the here shown example, the length of the critical period is 0.8-0.46 = 0.34. The slope of the indicator accurately indicates the future state, i.e. similarity is $>$ 0.99, during a fraction of 0.29/0.34 = 0.85 of this period. The full time series is shown in Fig. 2.

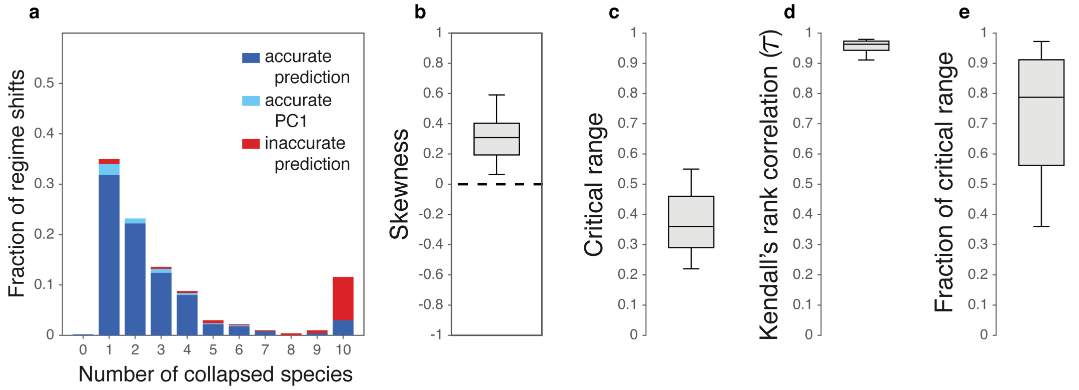

Figure S8 Overall statistics on the performance of the indicator when competitive interaction strengths, c_{ij} are taken from $\sim U(0.02, 0.08)$. **(a)** The performance of the indicator for different numbers of collapsed species. The fraction of regime shifts for which the change in abundance was not well indicated is shown in red. The fraction accurately indicated by the first principal component, but not by the direction in which time points are skewed is shown in light blue. Fully accurate predictions are indicated in dark blue. **(b)** The skewness of time points projected on the first principal component. A positive skewness means that time points were skewed in the direction of the networks future state. **(c)** The length of the critical range in which the indicator's magnitude increases significantly. **(d)** Kendall's rank correlation, τ, as determined for the critical range. **(e)** The fraction of the critical range in which the slope of the indicator accurately indicates the future state, i.e. in which the similarity between the first principal component and the observed shift in abundance is > 0.99. Results in panels (b-e) are shown for regime shifts that were accurately indicated by the first principal component. Box plots show the median and the upper and lower quartiles. Whiskers correspond to the 9th and the 91st percentile.

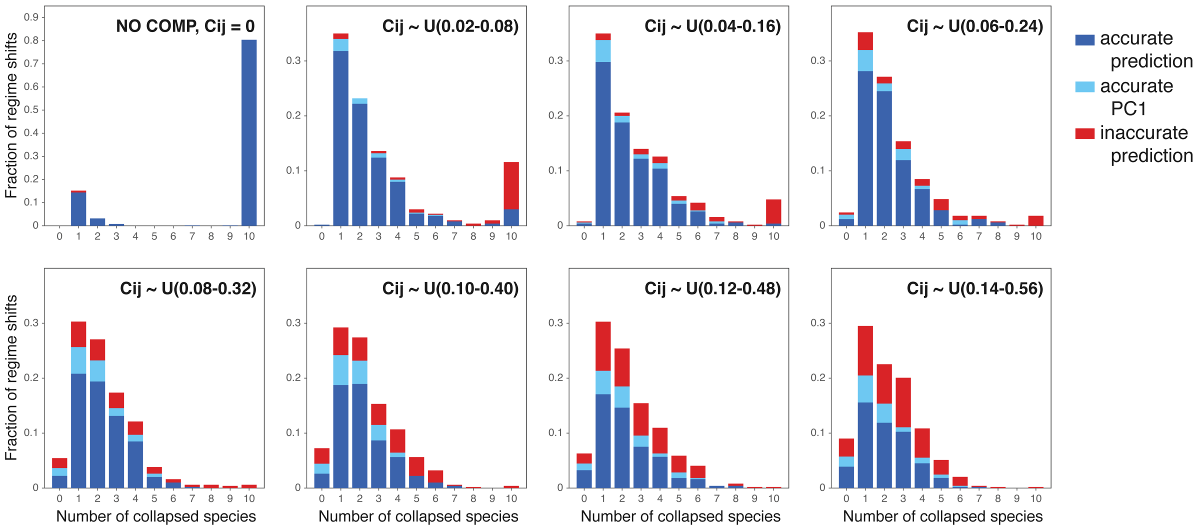

Figure S9 The number of pollinator species collapsing to extinction as observed in data sets of 1000 regime shifts. Each panel shows results when sampling competitive interaction strengths from a different parameter range (see ranges indicated). In the extreme case where there was no competition (top left panel), we found almost exclusively full network collapses (i.e. all ten pollinator species collapsed to extinction). As the strength of competition increases, full network collapses become less frequent. Partial network collapses tend to be small independent of the strength of competition, i.e. the most common partial collapse led to the extinction of only one single pollinator species. The fraction of regime shifts for which the change in abundance was not well indicated is shown in red. The fraction accurately indicated by the first principal component, i.e. the slope of the indicator is accurate, but not by the direction in which time points are skewed is shown in light blue. Fully accurate predictions are indicated in dark blue.

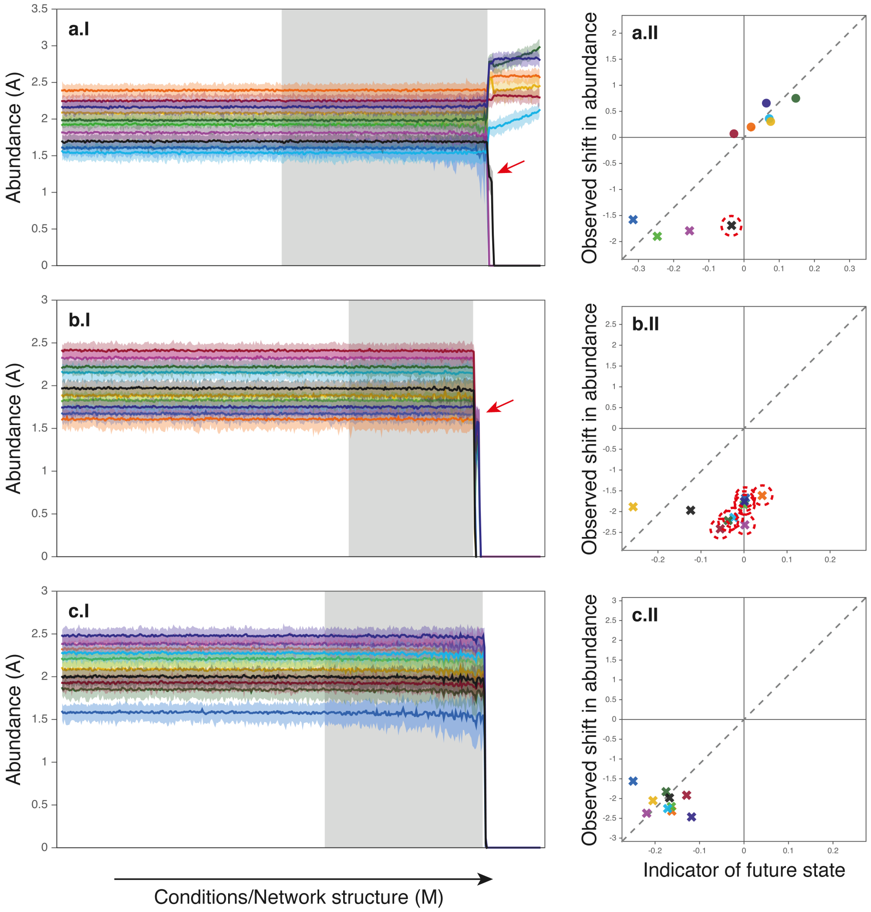

Figure S10 Two cascading collapses and one immediate collapse. **(a)** Example of a cascading collapse that eventually leads to the collapse of four pollinator species. Three species (blue, green and purple) collapse to extinction rapidly. A fourth (black) species collapses as well, but remains for a short while at a lower abundance before collapsing to extinction (red arrow, a.I). Out of the four species that collapse to extinction, the black species is also the one for which the indicated loss in abundance is smallest (red circle, a.II). **(b)** Example of a cascading collapse that eventually leads to a full collapse of the network (i.e. the most common outcome of a cascading collapse). Two species (black and yellow) collapse to extinction rapidly. The other species collapse as well, but remain for a short while at a lower abundance before collapsing to extinction (red arrow, b.I). The indicated loss in abundance of the rapidly collapsing species is much bigger than the loss indicated for the species that collapse a bit later (red circles, b.II). **(c)** Example of a full network collapse that was accurately indicated. All species collapse at approximately the same time (c.I). All species were indicated to lose in abundance (c.II).

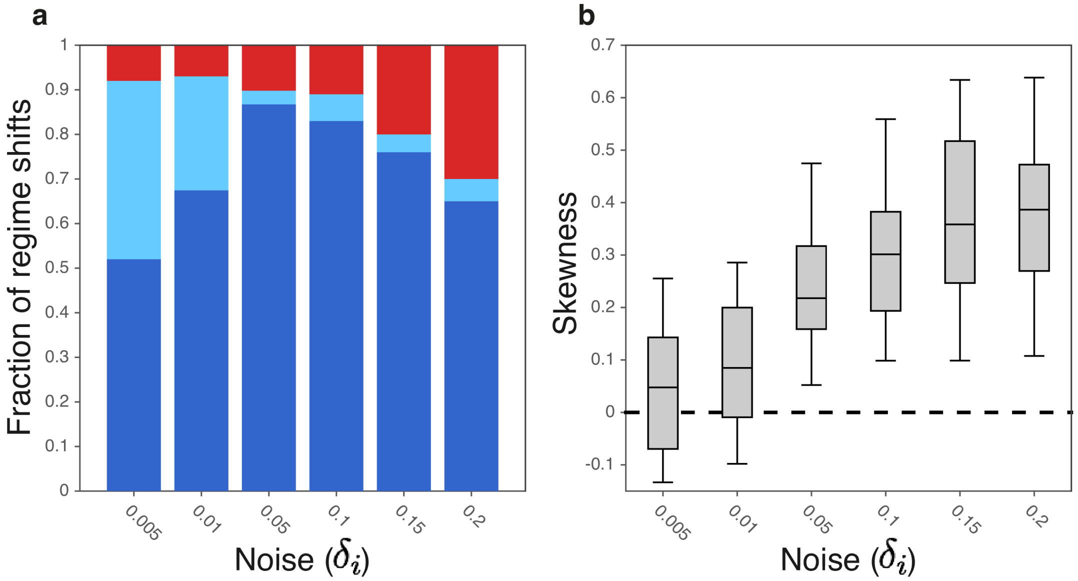

Figure S11 Performance of the indicator for different noise levels (noise levels, ϵ_i, are indicated on the x-axis). **(a)** The fraction of accurately indicated regime shifts (dark blue), the fraction accurately indicated by the first principal component, i.e. the slope of the indicator is accurate, but not by the direction in which time points are skewed (light blue), and the fraction of inaccurately indicated regime shifts (red). **(b)** The skewness of time points projected on the first principal component. A positive skewness means that time points are skewed in the direction of a network's future state. The skewness is shown for regime shifts that were accurately indicated by the first principal component.

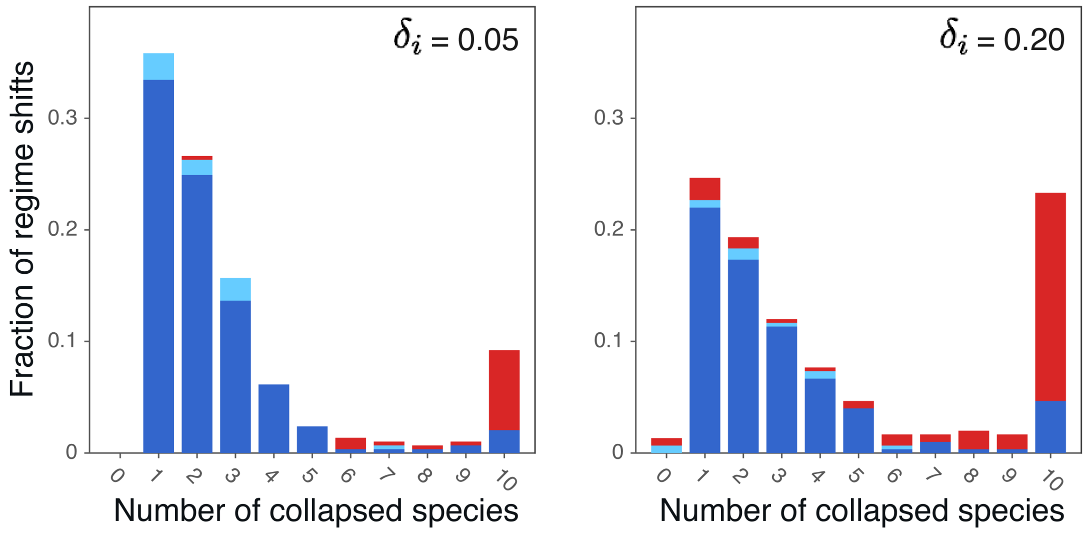

Figure S12 The number of pollinator species collapsing to extinction as observed in data sets of 1000 regime shifts when noise levels are low (left panel, $\epsilon_i = 0.05$) and when noise levels are high (left panel, $\epsilon_i = 0.2$). Full network collapses were found to occur more frequently when noise levels are high. The fraction of regime shifts for which the change in abundance was not well indicated is shown in red. The fraction accurately indicated by the first principal component, i.e. the slope of the indicator is accurate, but not by the direction in which time points are skewed is shown in light blue. Fully accurate predictions are indicated in dark blue.

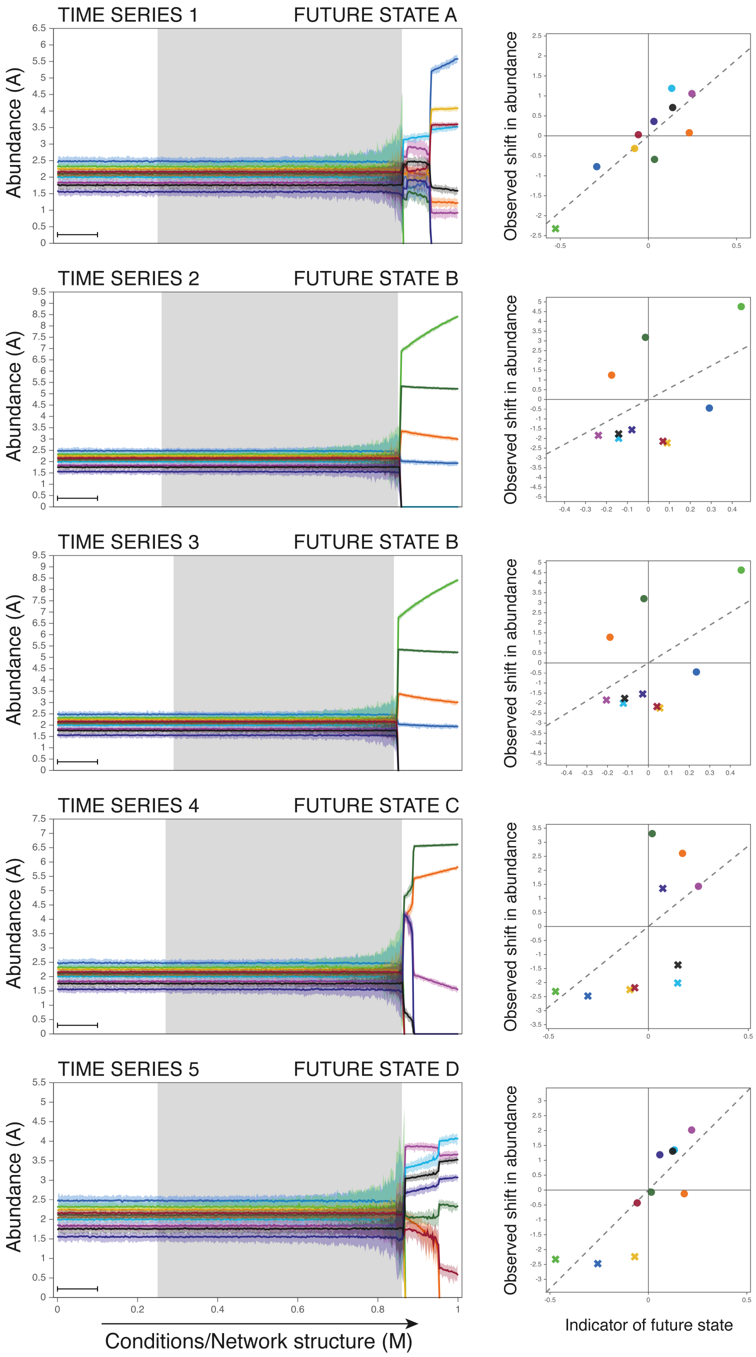

Figure S13 Five time series of a network that shows 'unpredictable' behavior. Even though the resilience of the network is undermined in the exact same way, the network may shift to several alternative future states. The future state of the network is determined by the only stochastic element in our model; the small-scale perturbations to which the network is permanently subjected. We found that this network may shift to (at least) four different future states (i.e. Euclidean distance between future states > 1.5). Of these future states, future state A and D are well indicated by the indicator (i.e. similarity > 0.99). The future state of the network is the same only in time series 2 and 3.

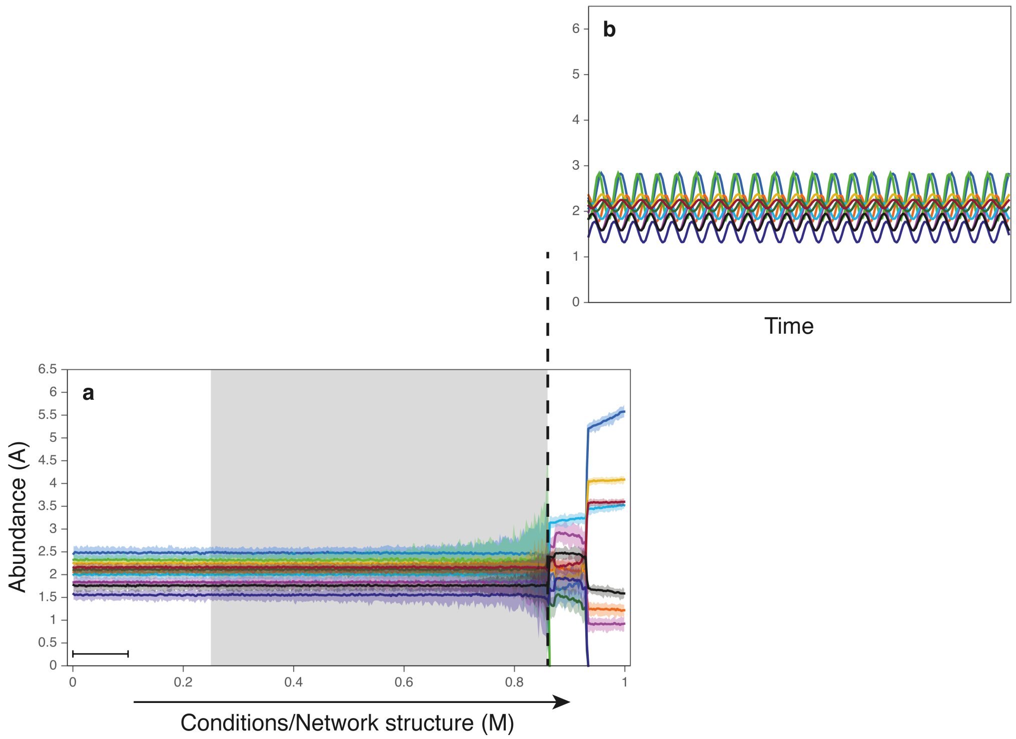

Figure S14 Example of a network approaching a supercritical Hopf bifurcation. **(a)** Time series of the network as conditions change. **(b)** Time series at fixed conditions just after the bifurcation point when assuming there are no external perturbations ($\epsilon = 0$). As can be seen from the dynamics we are dealing with a limit cycle. In the presence of external perturbations, the fluctuations caused by these dynamics are amplified and lead to a partial collapse of the network.

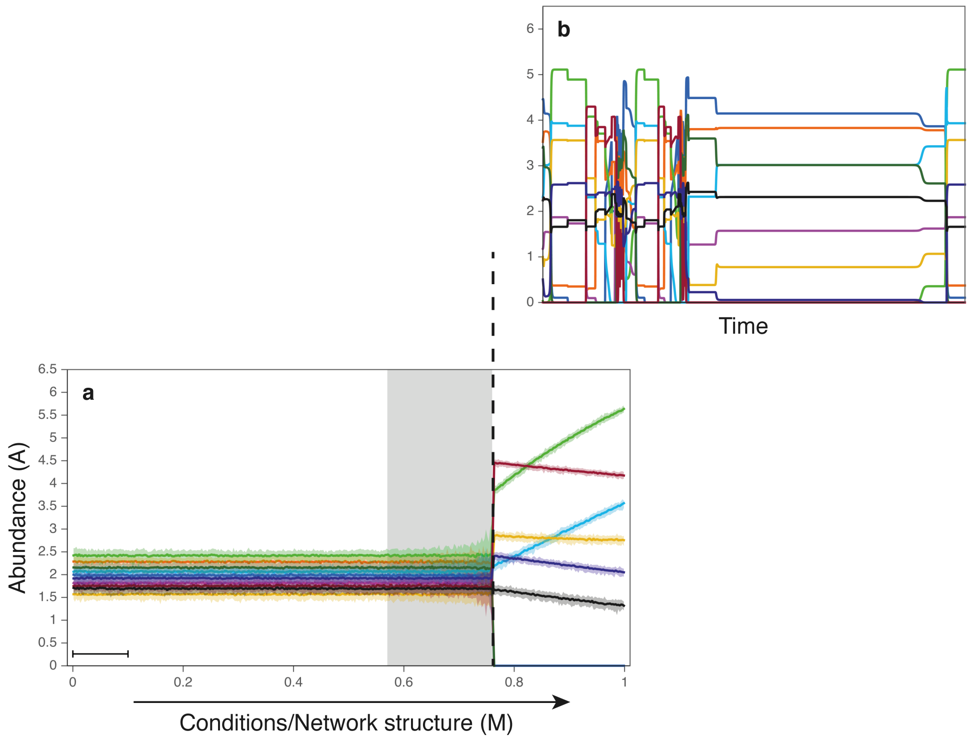

Figure S15 Example of a network approaching a subcritical Hopf bifurcation. **(a)** Time series of the network as conditions change. **(b)** Time series at fixed conditions just after the bifurcation point when assuming there are no external perturbations ($\epsilon = 0$) and when excluding the condition that populations of a size smaller than 0.001 have a zero growth rate (dN/dt=0). As can be seen from the dynamics we are dealing with chaotic/heteroclinic dynamics. The condition that populations of a size smaller than 0.001 have a zero growth rate leads to a partial collapse of the network. Which species are the first to cross this threshold is strongly influenced by the stochastic perturbations that are constantly disturbing the network.

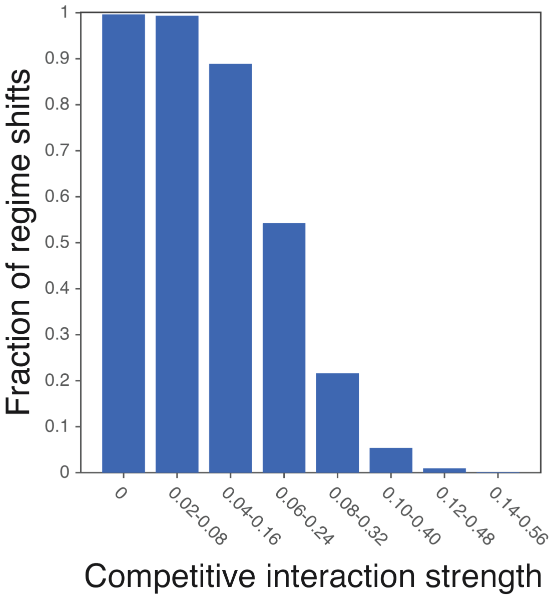

Figure S16 The probability of finding a stable solution at initial conditions, $M = 0$, when sampling competitive interaction strengths from different parameter ranges (ranges are indicated on the x-axis). As the strength of competition increases, it becomes increasingly difficult to find a stable solution. When there is no competition between species, the probability of finding a stable solution is nearly one. For the highest competition level we tested, i.e. (0.14,0.56), this probability was below 0.01. Results are shown for networks of 10 plants and 10 pollinators as described in Appendix S5.

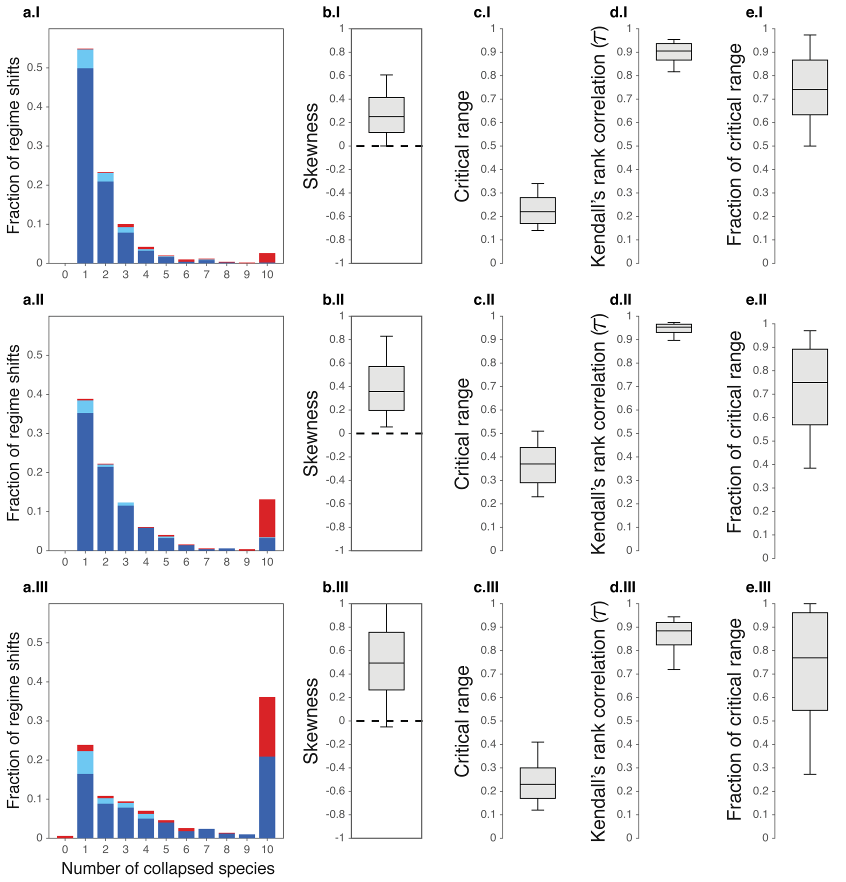

Figure S17 Overall statistics on the performance of the indicator when nontrivial equilibrium tend to increase **(I)**, change but stay the same on average **(II)**, and when abundances tend to decrease **(III)**. Results are shown for data sets of 1000 regime shifts. As in Fig. S8 we show: **(a)** the performance of the indicator for different numbers of collapsed species, **(b)** the skewness of time points projected on the first principal component, **(c)** the length of the critical range in which the indicator's magnitude increases significantly, **(d)** Kendall's rank correlation, τ, as determined for the critical range, and **(e)** the fraction of the critical range in which the slope of the indicator accurately indicates the future state. Box plots show the median and the upper and lower quartiles. Whiskers correspond to the 9th and the 91st percentile.

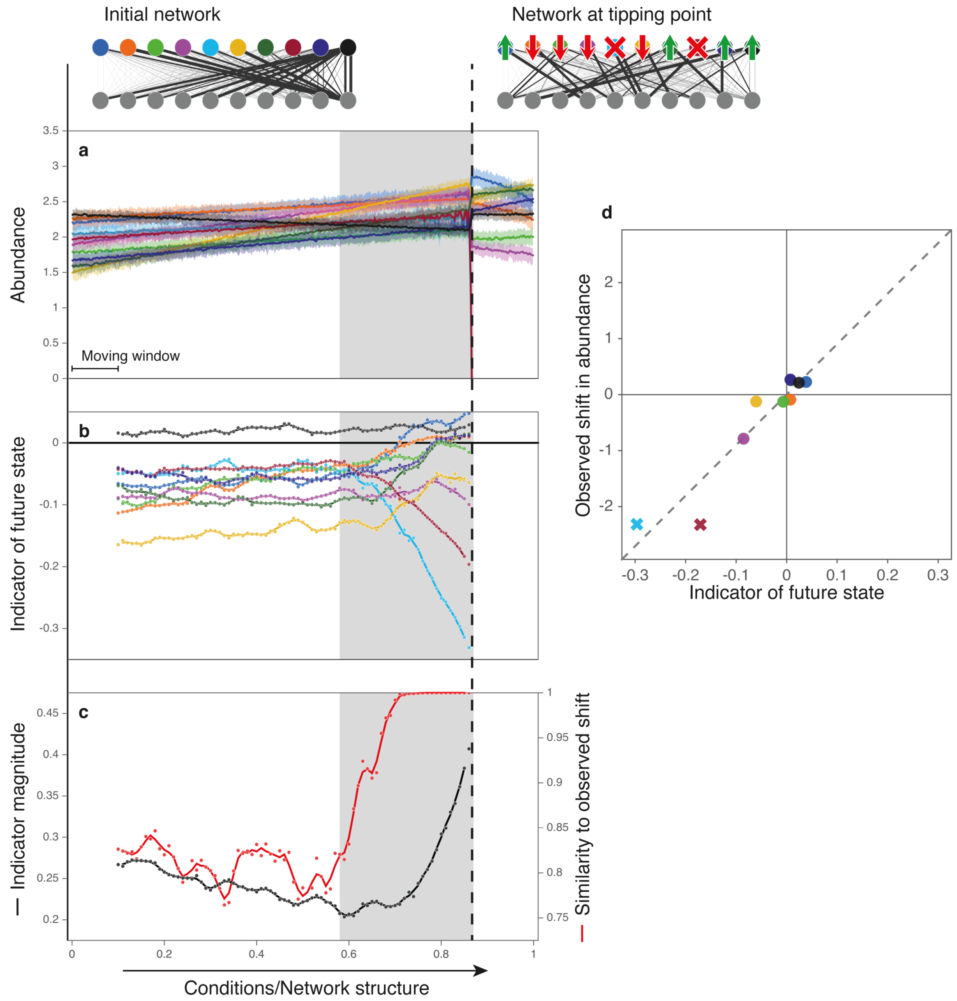

Figure S18 Directional slowing down when abundances tend to increase. **(a)** Time series of species belonging to one set of a bipartite mutualistic network, i.e. the pollinators. At the tipping point two species collapse to extinction (red and light blue). **(b)** The indicator of the future state measuring the direction in which fluctuations are distributed asymmetrically. **(c)** The magnitude of the indicator, reflecting the extent in which fluctuations are distributed asymmetrically, plotted together with the accuracy measured as the similarity between its direction and the observed shift in abundance. Grey bands indicate the period in which the indicators magnitude increases significantly. **(d)** The observed changes in abundance versus the scores on the indicator just before the tipping point. Extinct species are indicated with crosses. The initial network, at M=0, is the same as in Fig. 2.

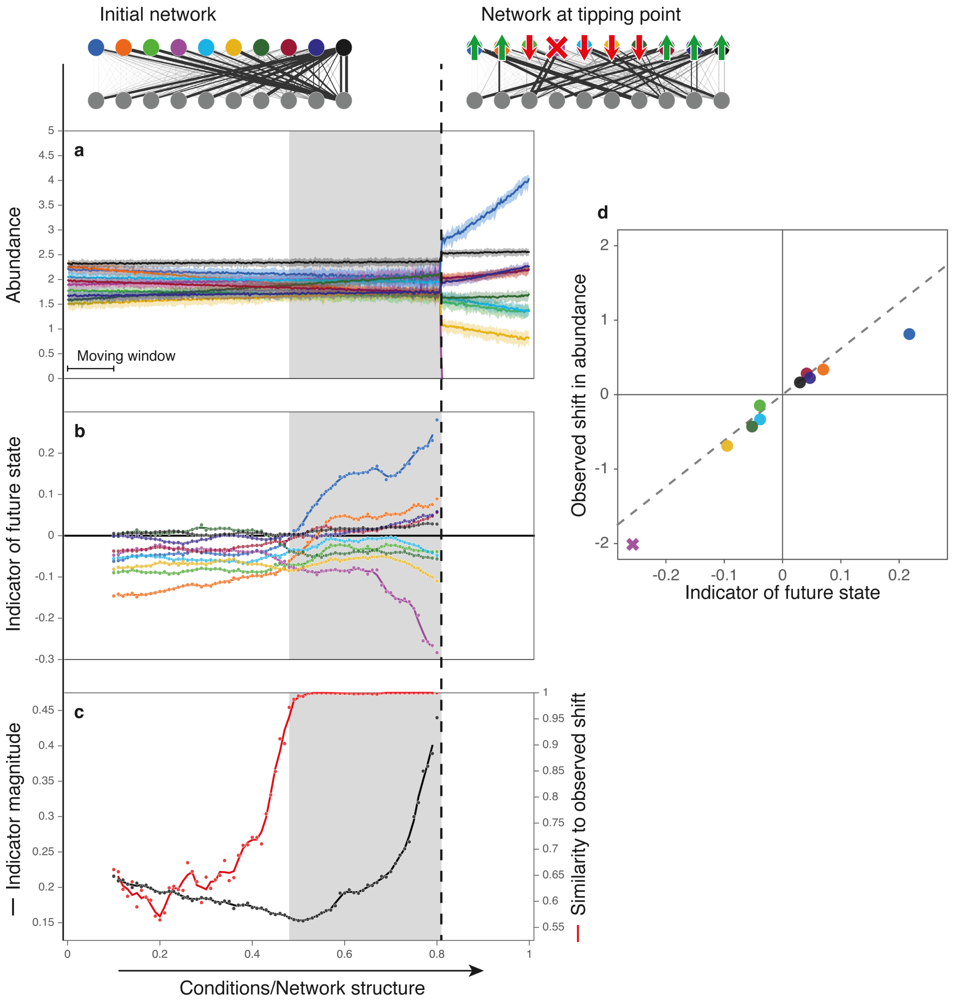

Figure S19 Directional slowing down when abundances change, but stay the same on average. **(a)** Time series of species belonging to one set of a bipartite mutualistic network, i.e. the pollinators. At the tipping point a single species collapses to extinction (purple). **(b)** The indicator of the future state measuring the direction in which fluctuations are distributed asymmetrically. **(c)** The magnitude of the indicator, reflecting the extent in which fluctuations are distributed asymmetrically, plotted together with the accuracy measured as the similarity between its direction and the observed shift in abundance. Grey bands indicate the period in which the indicators magnitude increases significantly. **(d)** The observed changes in abundance versus the scores on the indicator just before the tipping point. Extinct species are indicated with crosses. The initial network, at M=0, is the same as in Fig. 2 and Fig. S18.

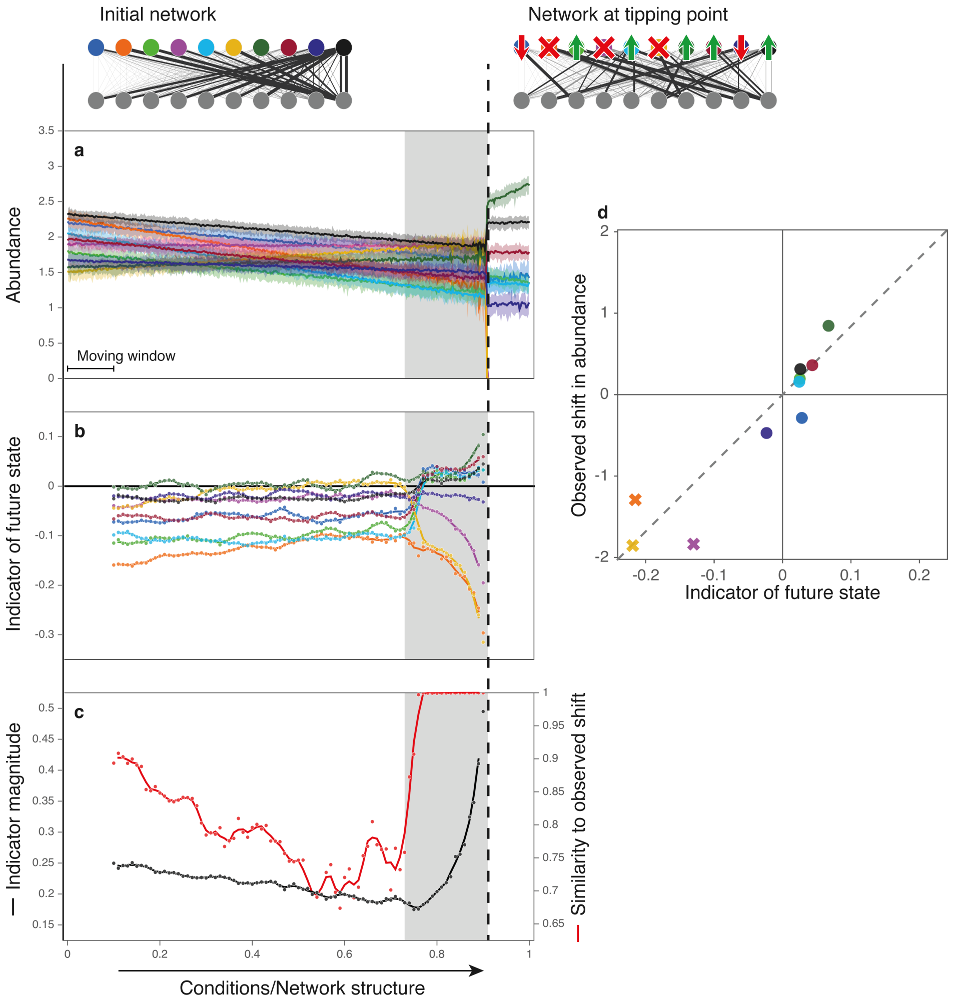

Figure S20 Directional slowing down when abundances tend to decrease. **(a)** Time series of species belonging to one set of a bipartite mutualistic network, i.e. the pollinators. At the tipping point three species collapse to extinction (yellow, purple and orange). **(b)** The indicator of the future state measuring the direction in which fluctuations are distributed asymmetrically. **(c)** The magnitude of the indicator, reflecting the extent in which fluctuations are distributed asymmetrically, plotted together with the accuracy measured as the similarity between its direction and the observed shift in abundance. Grey bands indicate the period in which the indicators magnitude increases significantly. **(d)** The observed changes in abundance versus the scores on the indicator just before the tipping point. Extinct species are indicated with crosses. The initial network, at M=0, is the same as in Fig. 2, Fig. S19, and Fig. S18.

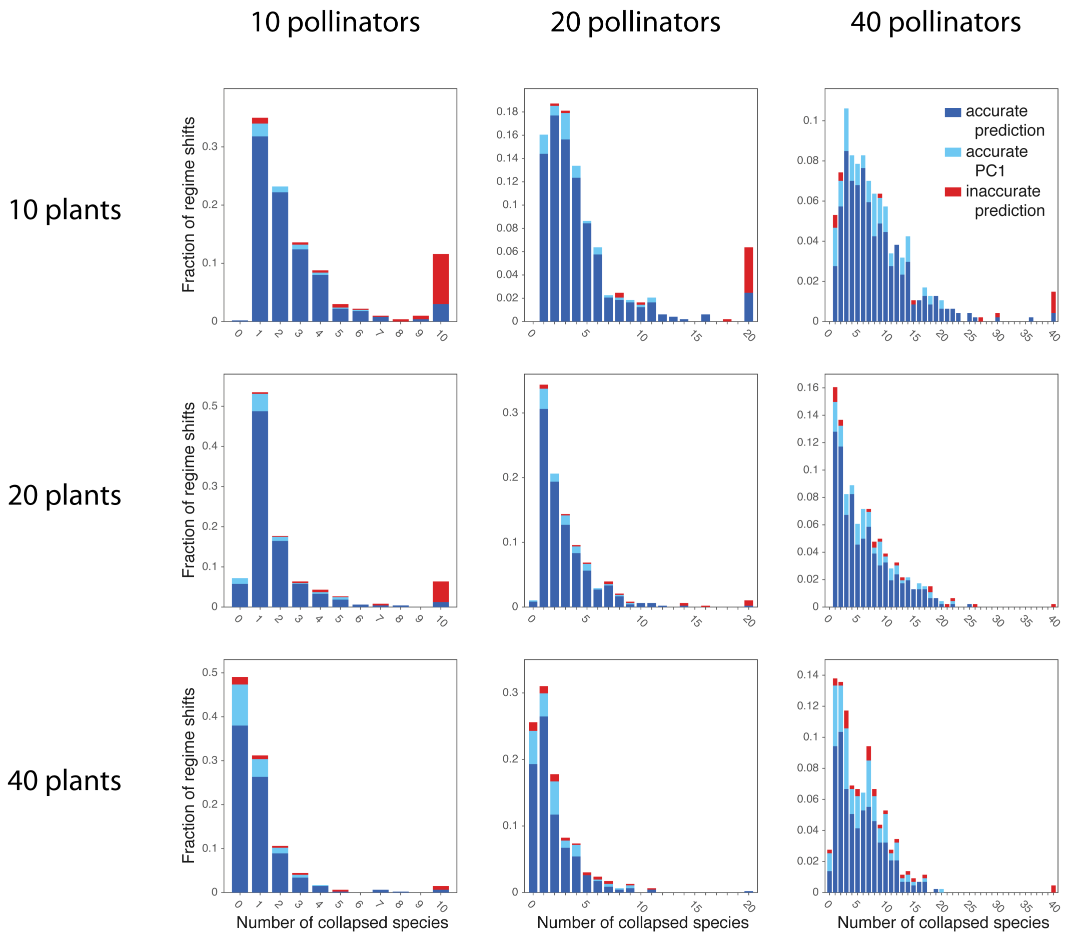

Figure S21 The performance of the indicator for networks of different size, i.e. for different number of plant (rows) and pollinator species (columns). Each panel shows the number of pollinator species collapsing to extinction as observed in data sets of 1000 regime shifts. The fraction of regime shifts for which the change in abundance was not well indicated is shown in red. The fraction accurately indicated by the first principal component, but not by the direction in which time points are skewed is shown in light blue. Fully accurate predictions are indicated in dark blue.

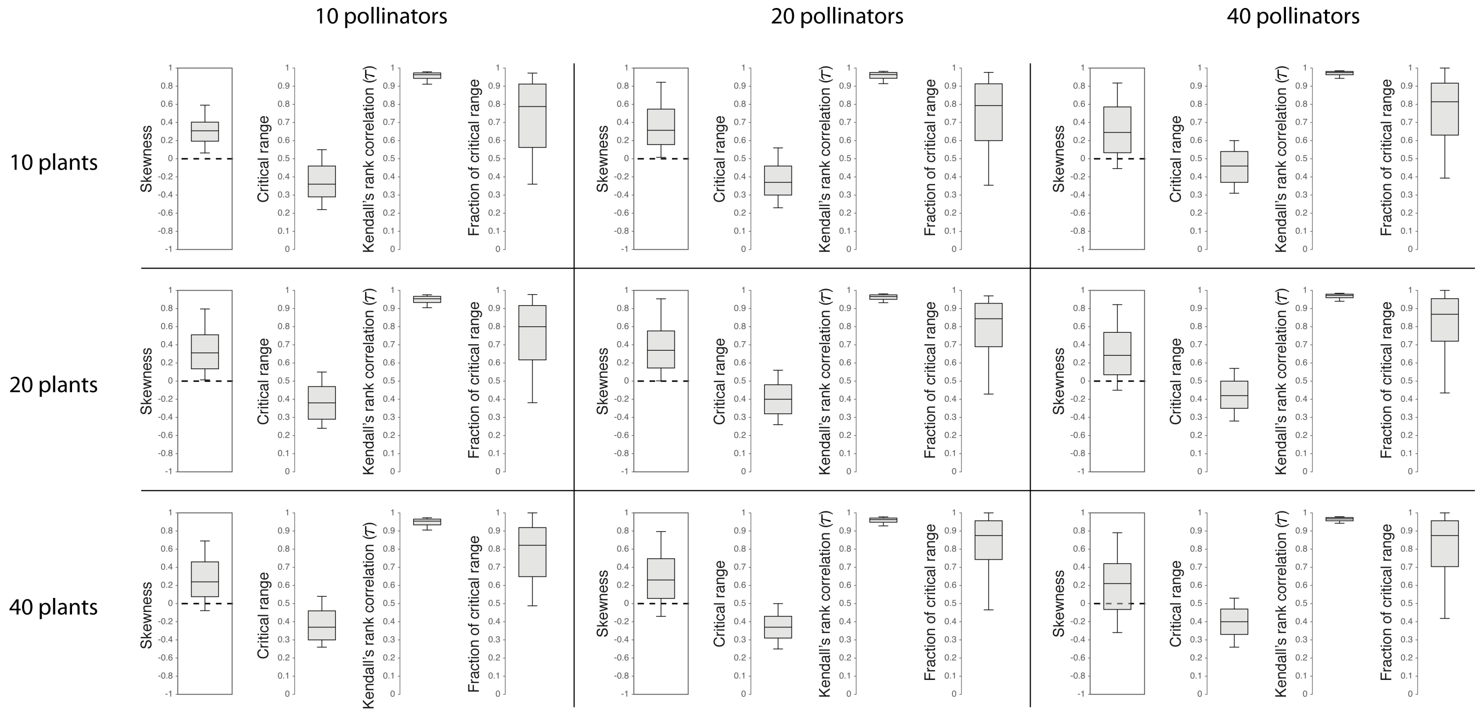

Figure S22 The performance of the indicator for networks of different size, i.e. for different number of plant (rows) and pollinator species (columns). Each panel shows the skewness of time points projected on the first principal component, the length of the critical range in which the indicator's magnitude increases significantly, Kendall's rank correlation, τ, as determined for the critical range, and the fraction of the critical range in which the slope of the indicator accurately indicates the future state. Results are shown for regime shifts that were accurately indicated by the first principal component. Results are shown for data sets of 1000 regime shifts. Box plots show the median and the upper and lower quartiles. Whiskers correspond to the 9th and the 91st percentile.

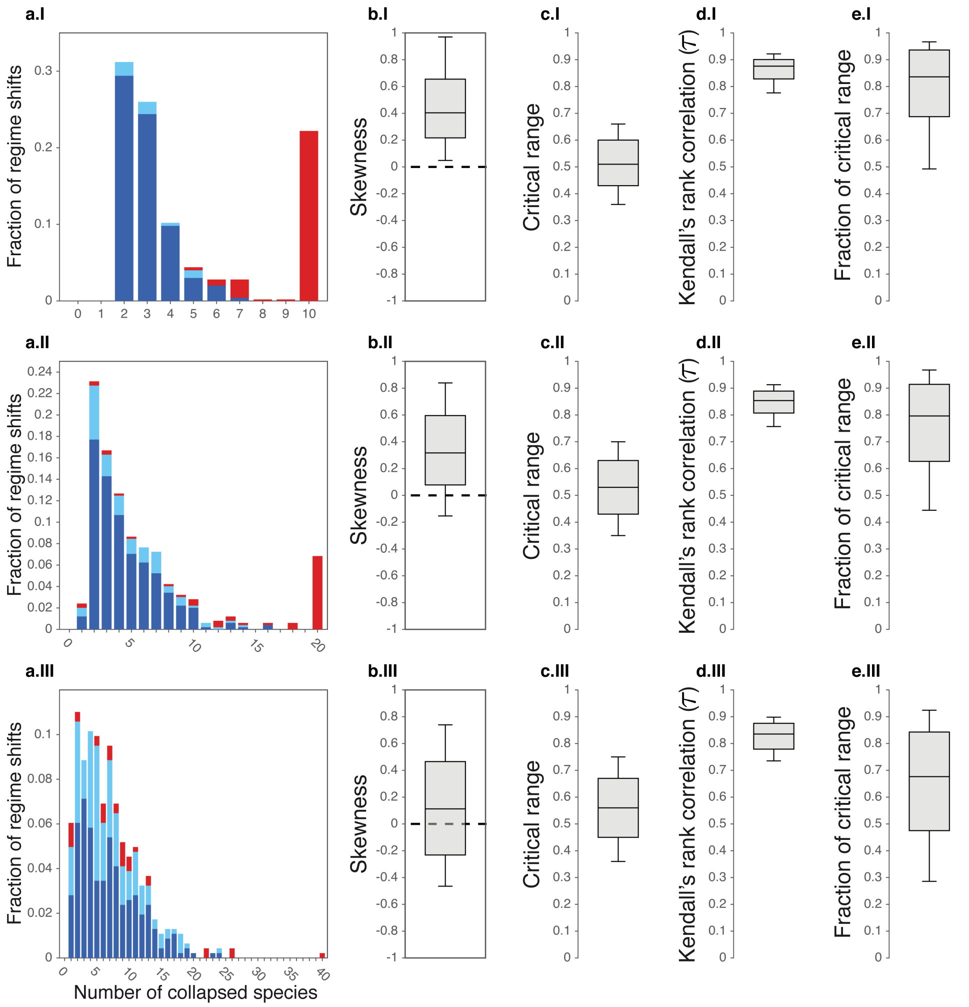

Figure S23 Overall statistics on the performance of the indicator when predicting the future state of a more general model of competition and facilitation. Results are shown for networks of 10 **(I)**, 20 **(II)** and 40 species **(III)**. **(a)** The performance of the indicator for different numbers of collapsed species. **(b)** The skewness of time points projected on the first principal component. **(c)** The length of the critical range in which the indicator's magnitude increases significantly. **(d)** Kendall's rank correlation, τ, as determined for the critical range. **(e)** The fraction of the critical range in which the slope of the indicator accurately indicates the future state, i.e. in which the similarity between the first principal component and the observed shift in abundance is > 0.99. Results in panels (b-e) are shown for regime shifts that were accurately indicated by the first principal component. Box plots show the median and the upper and lower quartiles. Whiskers correspond to the 9th and the 91st percentile.

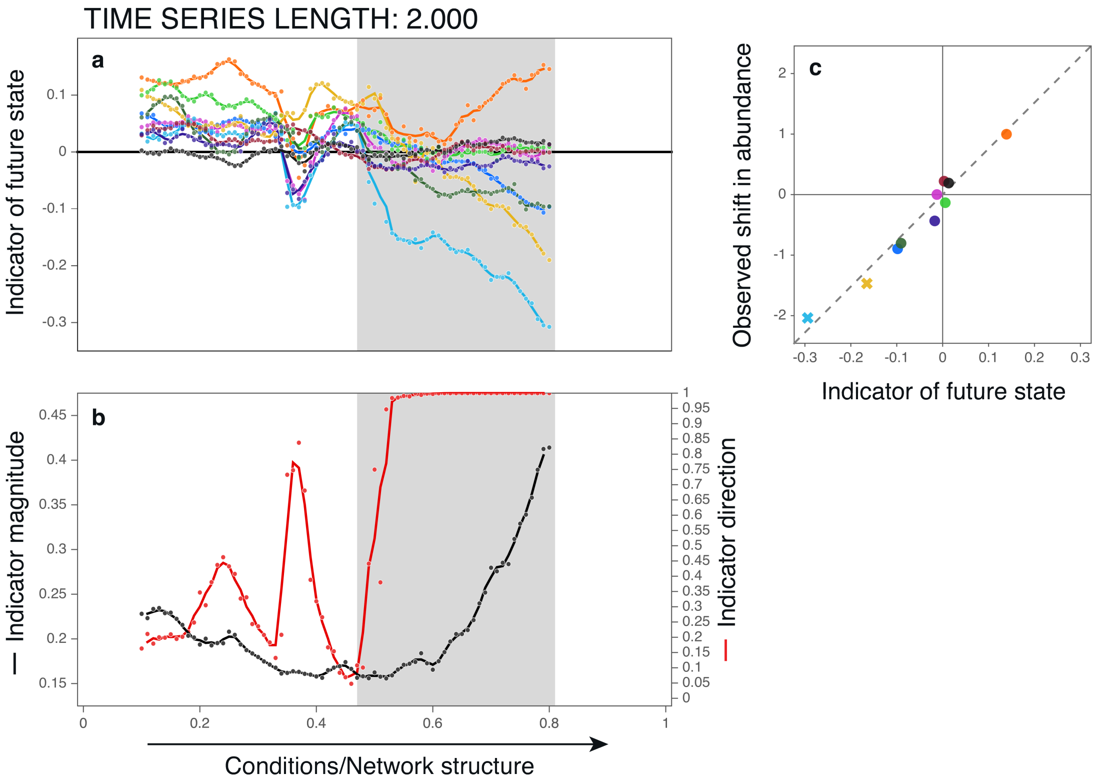

Figure S24 Directional slowing down as detected by the indicator when the total length of a time series is 2.000 time steps. **(a)** The indicator of the future state measuring the direction in which fluctuations are distributed asymmetrically. **(b)** The magnitude of the indicator, reflecting the extent in which fluctuations are distributed asymmetrically, plotted together with the accuracy measured as the similarity between its direction and the observed shift in abundance. Grey bands indicate the period in which the indicators magnitude increases significantly. **(c)** The observed changes in abundance versus the scores on the indicator just before the tipping point. Extinct species are indicated with crosses. The initial network, at $M = 0$, and the way in which this network is affected by changing environmental conditions, M, is the same as in Fig. 2. Changes in the direction and magnitude of the indicator are determined with a rolling window of 10% of the entire time series, i.e. 200 out of 2.000 time steps.

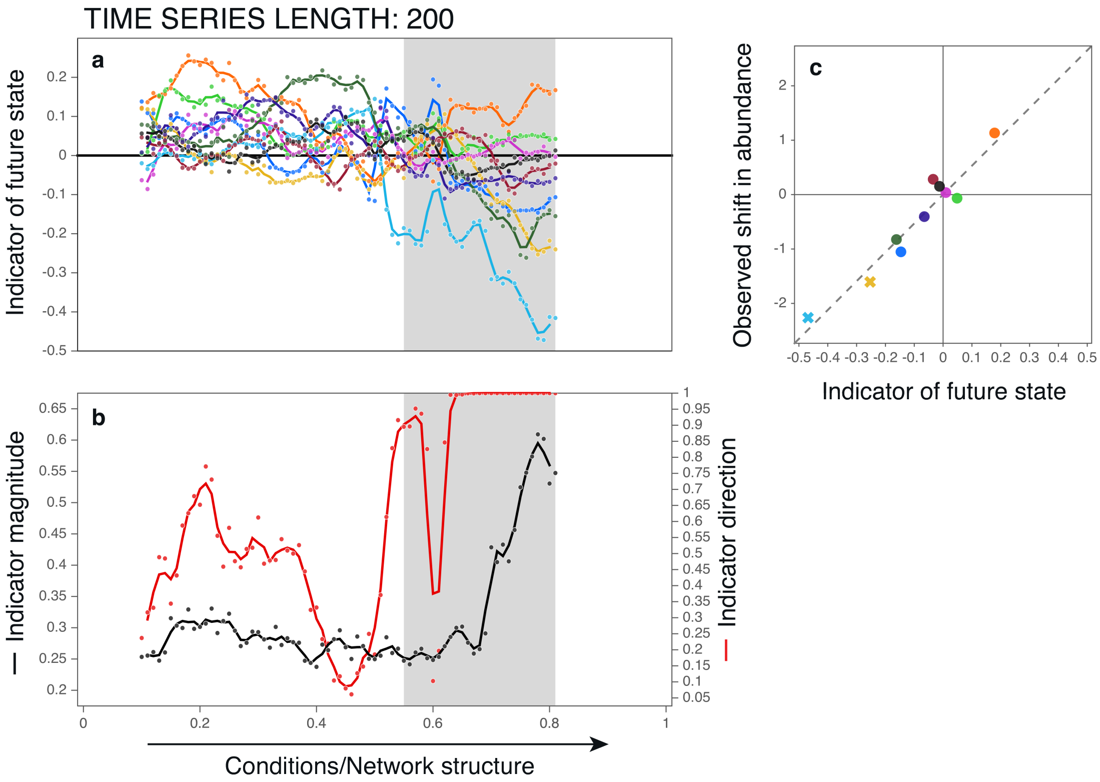

Figure S25 Directional slowing down as detected by the indicator when the total length of a time series is 200 time steps. **(a)** The indicator of the future state measuring the direction in which fluctuations are distributed asymmetrically. **(b)** The magnitude of the indicator, reflecting the extent in which fluctuations are distributed asymmetrically, plotted together with the accuracy measured as the similarity between its direction and the observed shift in abundance. Grey bands indicate the period in which the indicators magnitude increases significantly. **(c)** The observed changes in abundance versus the scores on the indicator just before the tipping point. Extinct species are indicated with crosses. The initial network, at $M = 0$, and the way in which this network is affected by changing environmental conditions, M, is the same as in Fig. 2. Changes in the direction and magnitude of the indicator are determined with a rolling window of 10% of the entire time series, i.e. 20 out of 200 time steps.

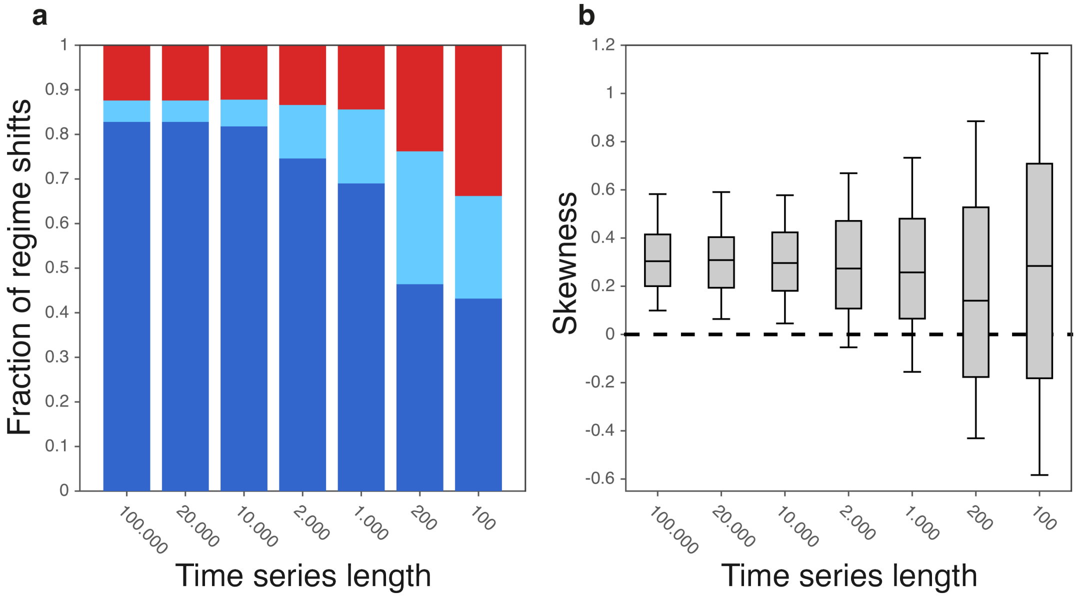

Figure S26 Performance of the indicator when time series have a different length (lengths are indicated on the x-axis). **(a)** The fraction of accurately indicated regime shifts (dark blue), the fraction accurately indicated by the first principal component, i.e. the slope of the indicator is accurate, but not by the direction in which time points are skewed (light blue), and the fraction of inaccurately indicated regime shifts (red). **(b)** The skewness of time points projected on the first principal component. A positive skewness means that time points are skewed in the direction of a network's future state. The skewness is shown for regime shifts that were accurately indicated by the first principal component. Changes in the direction and magnitude of the indicator are determined with a rolling window of 10% of the entire time series

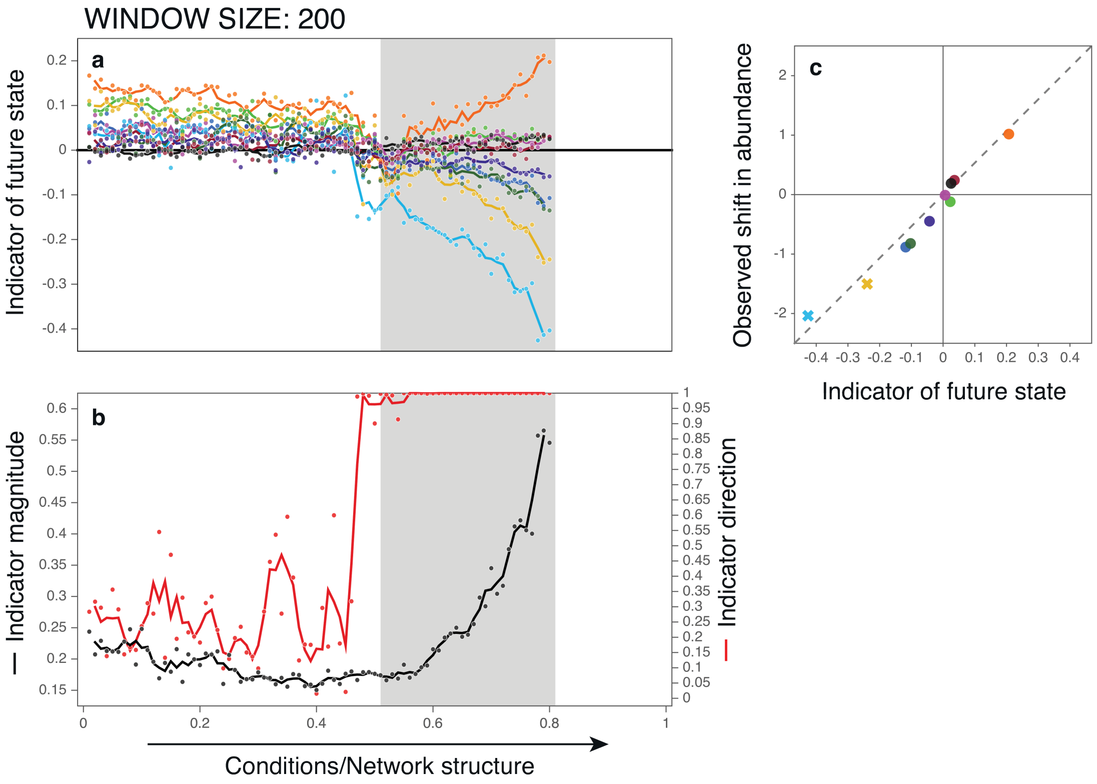

Figure S27 Directional slowing down as detected by the indicator when using a rolling window of 1% of the entire time series, i.e. 200 out of 20.000 time steps. **(a)** The indicator of the future state measuring the direction in which fluctuations are distributed asymmetrically. **(b)** The magnitude of the indicator, reflecting the extent in which fluctuations are distributed asymmetrically, plotted together with the accuracy measured as the similarity between its direction and the observed shift in abundance. Grey bands indicate the period in which the indicators magnitude increases significantly. **(c)** The observed changes in abundance versus the scores on the indicator just before the tipping point. Extinct species are indicated with crosses. The initial network, at $M = 0$, and the way in which this network is affected by changing environmental conditions, M, is the same as in Fig. 2.

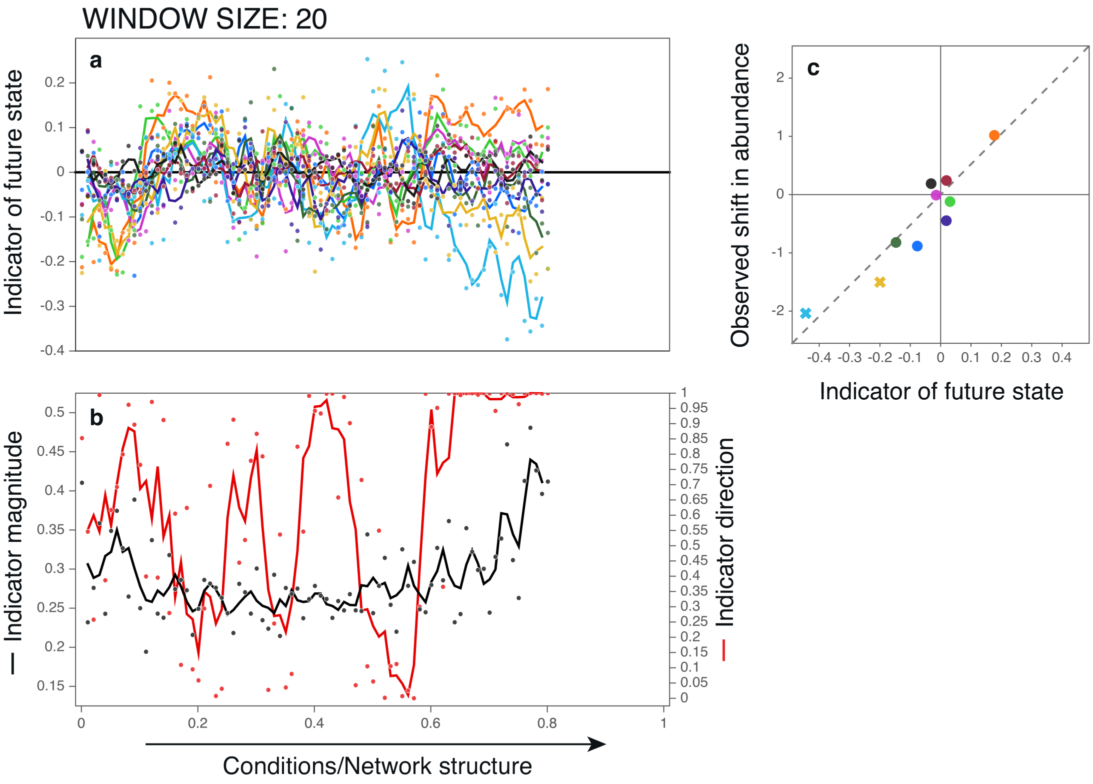

Figure S28 Directional slowing down as detected by the indicator when using a rolling window of 0.1% of the entire time series, i.e. 20 out of 20.000 time steps. **(a)** The indicator of the future state measuring the direction in which fluctuations are distributed asymmetrically. **(b)** The magnitude of the indicator, reflecting the extent in which fluctuations are distributed asymmetrically, plotted together with the accuracy measured as the similarity between its direction and the observed shift in abundance. No significant increase in the indicator's magnitude was detected. **(c)** The observed changes in abundance versus the scores on the indicator just before the tipping point. Extinct species are indicated with crosses. The initial network, at $M = 0$, and the way in which this network is affected by changing environmental conditions, M, is the same as in Fig. 2.

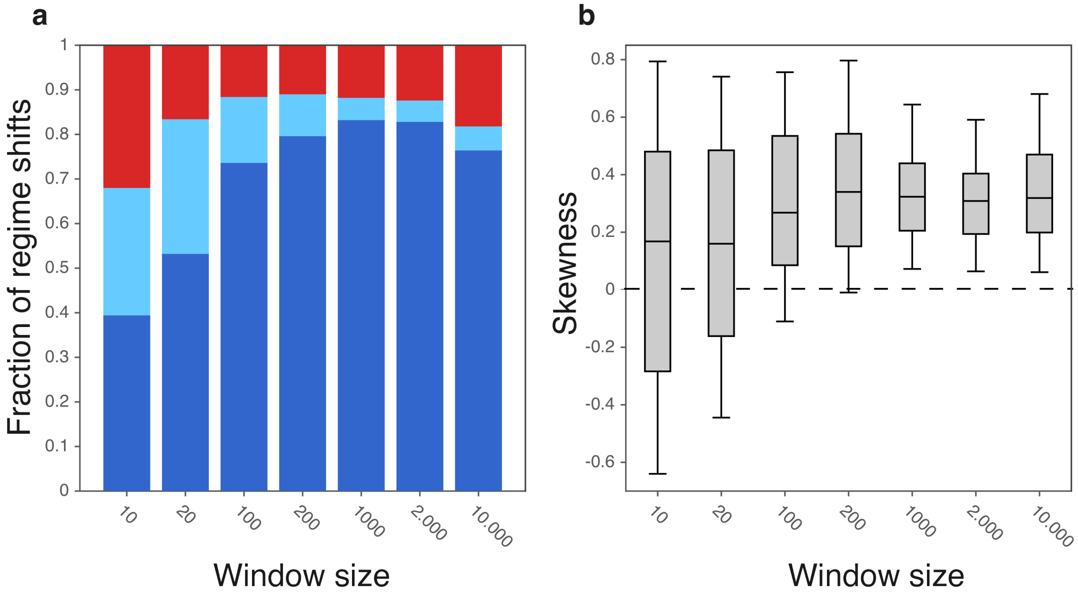

Figure S29 Performance of the indicator when the rolling window has a different length (lengths are indicated on the x-axis). **(a)** The fraction of accurately indicated regime shifts (dark blue), the fraction accurately indicated by the first principal component, i.e. the slope of the indicator is accurate, but not by the direction in which time points are skewed (light blue), and the fraction of inaccurately indicated regime shifts (red). **(b)** The skewness of time points projected on the first principal component. A positive skewness means that time points are skewed in the direction of a network's future state. The skewness is shown for regime shifts that were accurately indicated by the first principal component. Time series have a length of 20.000 time steps.

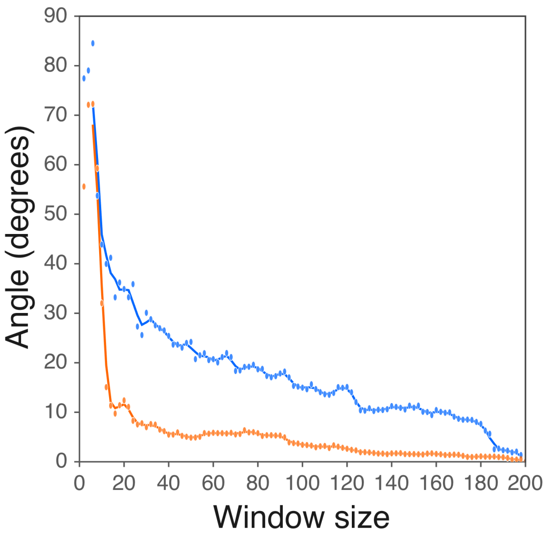

Figure S30 The extend in which the size of a rolling window affects the slope indicated by the first principal component far from a tipping point (blue) and close to a tipping point (orange). The y-axis corresponds to the difference in angle between the first principal component obtained for a window of 200 observations and for a window containing the number of observations indicated on the x-axis. The effect of an increasingly small window size on the direction of the first principal component is, in this example, much smaller close to a tipping point. Results are shown for the time series in Fig. 2.A at M=0.1 (blue) and M=0.78 (orange).
